# 4‑Fluorophenyl Tagging as a Diagnostic Tool for Monitoring Sulfur Oxidation States via ^19^F NMR

**DOI:** 10.1021/acs.joc.6c00917

**Published:** 2026-07-22

**Authors:** Makayla L. Williams, Twinkle I. Patel, Matthew J. Moschitto

**Affiliations:** Department of Medicinal Chemistry, 15484 Ernest Mario School of Pharmacy, Rutgers, the State University of New Jersey, 163 Frelinghuysen Road, Piscataway, New Jersey 08854, United States

## Abstract

We report a simple and general strategy to monitor sulfur oxidation states using ^19^F NMR by incorporating a 4-fluorophenyl tag on sulfur-containing molecules. A series of structurally related compounds spanning various sulfur oxidation states were compiled, and their ^19^F NMR chemical shifts were recorded to establish a diagnostic fingerprint tool. This approach enables rapid characterization of products and byproducts in sulfur-based transformations, particularly those involving redox changes such as disproportionation. To demonstrate the utility of the method, we applied it to study reactions involving sulfur oxidation state changes, establishing a proof-of-concept for this diagnostic platform. This technique offers a valuable tool for mechanistic analysis and product profiling in diverse areas of sulfur chemistry.

## Graphical abstract

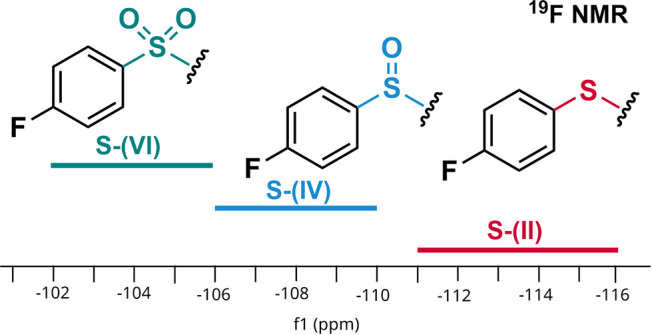

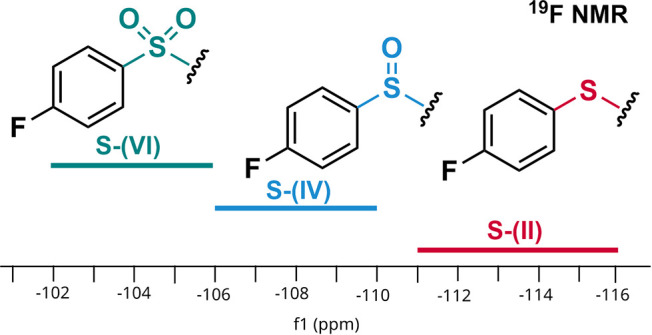

## Introduction

Organosulfur functionalities are found in numerous value-added products including pharmaceuticals, polymers and materials.^1–3^ Sulfur is present in more than one-fifth of FDA-approved drugs, where it frequently influences membrane permeability, metabolic stability, and target affinity (Figure 1a).^4,5^ The oxidation state of sulfur in a drug molecule directly affects its reactivity, electronic properties, and biological profile. Thioethers, for instance, are commonly used to modulate lipophilicity and can serve as masked leaving groups or metabolic soft spots. Sulfoxides introduce polarity and chirality, as seen in the proton pump inhibitor esomeprazole, where the sulfoxide stereochemistry governs therapeutic efficacy.^6^ Sulfones and sulfonamides are electron-deficient and metabolically stable, and are often used to enhance aqueous solubility and engage in hydrogen bonding; representative examples include dapsone (a sulfone antibiotic) and celecoxib (a sulfonamide-based COX-2 inhibitor).^7–10^ Sulfonic acids, such as in furosemide, contribute strong acidity and water solubility and are often employed in prodrug or ionizable motifs. From a synthetic standpoint, sulfinates salts,^11,12^ sulfonyl fluorides,^13,14^ and sulfinamides serve a precursors to more complex sulfur-(VI) functionalities.^15^

**Figure 1:**
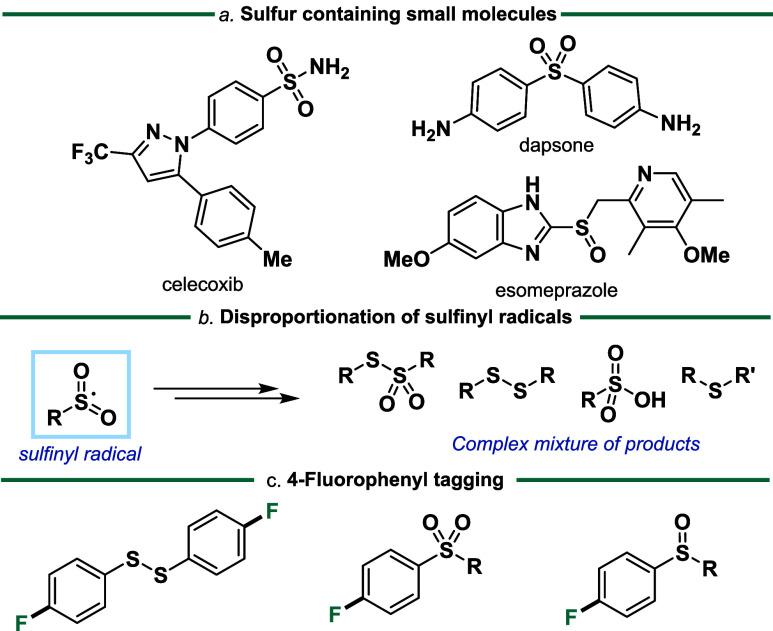
Sulfur containing small molecules and common disproportionation products.

Importantly, synthetic access to these functional groups often involves redox interconversion between sulfur oxidation states, underscoring the need for reliable analytical techniques to monitor such changes during reaction development. Previous efforts in our laboratory have focused on the formation of sulfinyl radicals from sulfinate salts and *N*-hydroxymethylphthalimide precursors.^16,17^ One deleterious process common with sulfinyl radicals is their tendency to disproportionate. Disproportionation arises from a complex rearrangement involving intermediates that form after the dimerization of sulfinyl radicals (Figure 1b).^18–20^ This leads to common byproducts including disulfides, thiosulfonates, sulfides, sulfones, sulfonic acids, and thiols. Rapid quantification of these byproducts is often difficult.

Unambiguous differentiation of sulfur oxidation states, especially in complex mixtures, remains a challenge with conventional spectroscopic and spectrometric techniques. Typically, reaction optimization is performed via ^1^H NMR, GC/MS, or LC/MS. While these methods are effective for detecting product formation, each has inherent limitations. ^1^H NMR requires well-resolved, isolated signals and careful selection of an internal standard that does not overlap with peaks in a crude reaction mixture. LC/MS and GC/MS both depend on efficient ionization and analyte stability. In our experience, gentle ionization methods such as electrospray (ESI) used in LC/MS do not reliably ionize sulfur-(VI) species, whereas harsher methods such as electron-impact (EI) used in GC/MS often lead to extensive fragmentation rather than intact molecular ions. As a result, it can be difficult to distinguish between sulfur-(II) products and fragmented sulfur-(VI) species.

^19^F NMR has long been employed as a method for tracking reaction progress and obtaining yields in organic synthesis.^21–25^ In an effort to streamline our analysis of sulfur-based reactions, we turned to ^19^F NMR as a rapid method for the assignment of sulfur oxidation states within the reaction mixture, while also providing reliable quantitative yields that allow for direct comparison of reaction conditions without the need for product isolation. Herein, we propose the use of 4-fluorophenyl tags (Figure 1c) to monitor sulfur oxidation state and characterize products and byproducts associated with sulfur transformations through ^19^F NMR.^26^

## Results and Discussion

### Generation of a Diagnostic Fingerprint

A series of structurally related compounds containing the 4-fluorophenyl tag and various sulfur oxidation states were compiled, and their ^19^F NMR chemical shifts were recorded to establish a diagnostic fingerprint tool (Figure 2; see Table S1 for references). Sulfur-(VI)-containing molecules with the 4-fluorophenyl tag are concentrated from δ −102 ppm to −106 ppm, with C­(sp^2^)-S bonds having a more negative shift than C­(sp^3^)-S bonds. Sulfur-(IV)-containing compounds are shifted further downfield, spanning from δ −106 ppm to −109 ppm. Sulfides, disulfides, thiols, and thioesters are clustered more broadly from δ −111 ppm to −116 ppm. The differences in chemical shifts observed for various sulfur oxidation states indicate that ^19^F NMR can be used as a diagnostic tool to monitor reaction progress involving interconversion between oxidation states.

**Figure 2:**
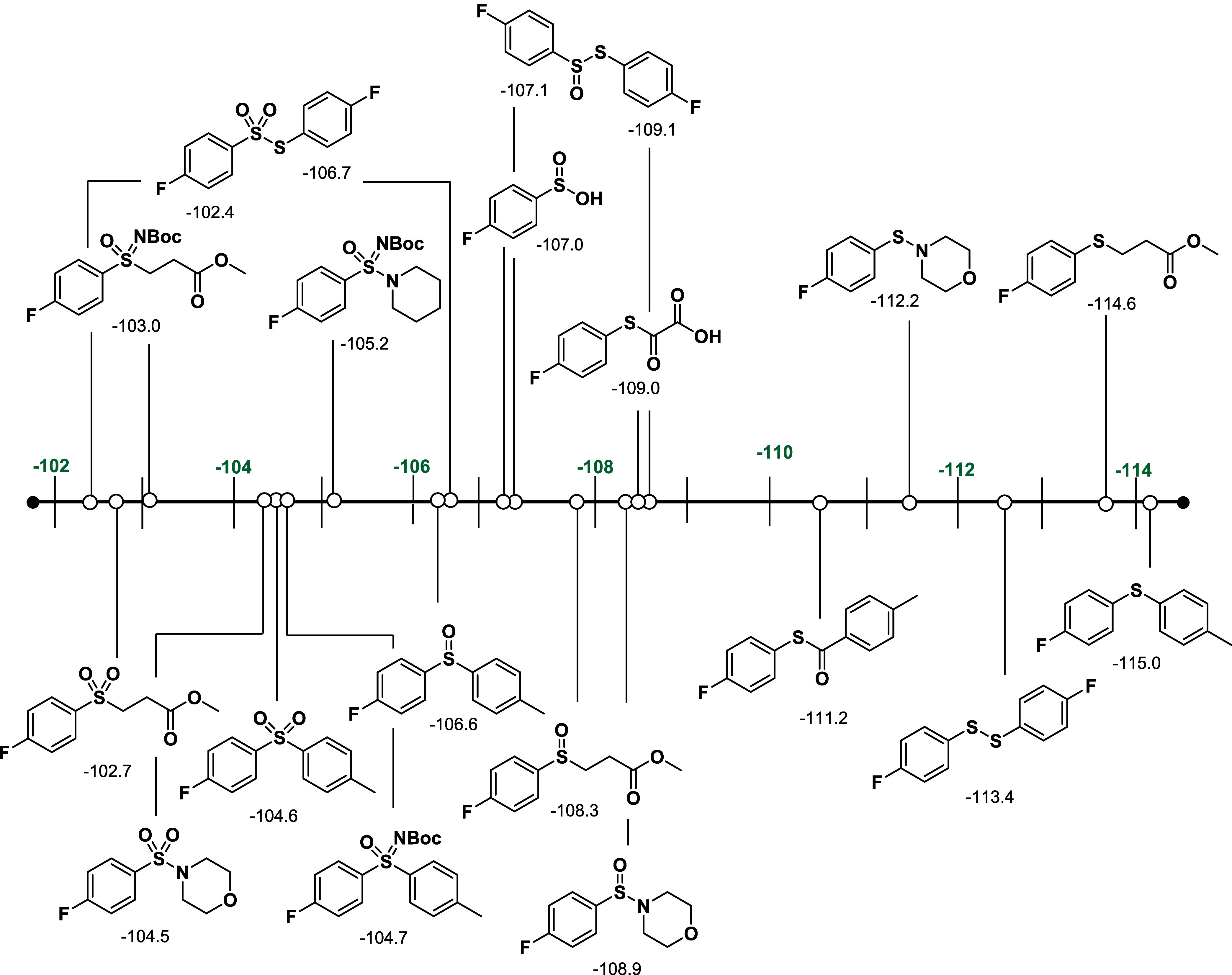
Known ^19^F chemical shifts of 4-fluorophenyl sulfur containing molecules. Units in ppm. See SI Table S1 for references.

To demonstrate this, the oxidation of sulfide **1a** to *N*-hydroxymethylphthalimide (NHMP) sulfone **1c** with *m*CPBA was monitored using ^19^F NMR (Figure 3). The change in shift observed over the course of this oxidation demonstrates that ^19^F NMR can monitor the interconversion of sulfur oxidation states containing the 4-fluorophenyl tag. Furthermore, as with many standard ^19^F methods, this method can be used for quantification of the individual components. While similar methods such as GC or LC allow for quantification at low concentrations, ^19^F does operate with a higher limit of detection, which is dependent on each operator’s NMR instrument and probe. In this study, we are able to detect samples in the low millimolar range with a 400 MHz NMR and BBO probe (see Supporting Information for limit of detection and quantification).

**Figure 3:**
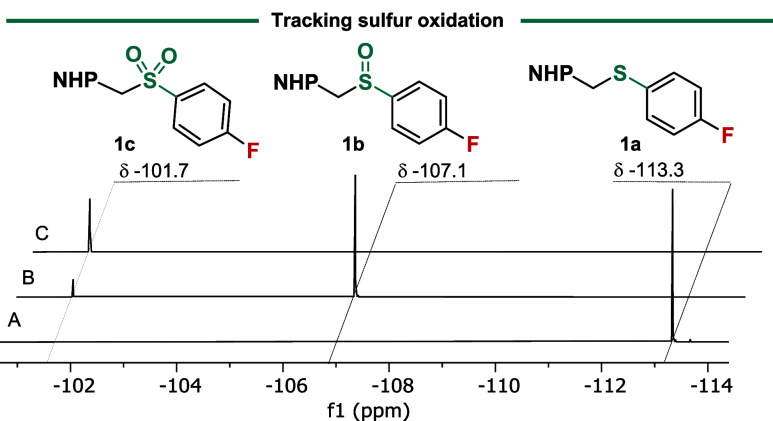
Oxidation state monitoring from sulfide to sulfone using ^19^F NMR (400 mHz, CDCl_3_). From sulfide **1a** (A, δ −113.3 ppm), 1.2 equiv of *m*CPBA was added. After stirring for 4 h, the mixture converted to sulfoxide **1b** (B, δ −107.1 ppm) with a small amount of sulfone present. Adding a second equivalent of oxidant resulted in full conversion to the sulfone **1c** (C, δ −101.7 ppm). NHP = *N*-hydroxyphthalimide.

### Probing *T*_1_ Relaxation Delays

In quantitative NMR (qNMR), accurate signal integration requires consideration of the longitudinal relaxation time (*T*_1_), which varies among nuclei and is influenced by factors such as solvent, pH, salt concentration, oxygen content, and magnetic field strength.^24^ To ensure reliable quantification, the repetition time (relaxation delay plus acquisition time) should be at least five to seven times the longest *T*_1_ value of the signals being analyzed, corresponding to approximately 99.0–99.9% signal recovery.^19^ Insufficient relaxation delays can lead to incomplete spin relaxation between scans, resulting in systematic integration errors and inaccurate concentration measurements.

To assess the impact of longitudinal relaxation on quantitative accuracy within our system, we measured *T*_1_ values for a representative set of eight molecules (Table 1). These measurements revealed that sulfur-(II) species exhibit longer *T*_1_ relaxation times compared to sulfur-(IV) and sulfur-(VI) analogues.

**Table 1:**
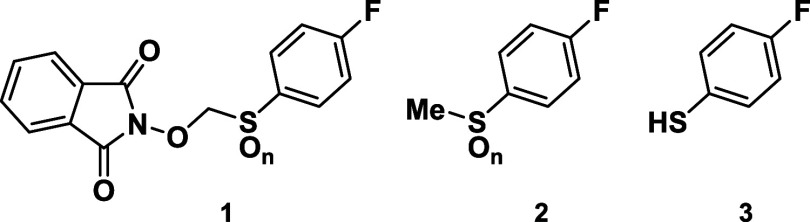
*T*_1_ Relaxation Delays for Sulfur Species

compound	*n*	^19^F shift (ppm)	*T*_1_ (s)
**1a**	0	–113.3	3.920
**1b**	1	–107.0	2.364
**1c**	2	–101.7	2.357
**2a**	0	–117.4	8.279
**2b**	1	–108.3	5.573
**2c**	2	–103.6	5.291
**3**	4-F PhSH	–116.6	5.865
Int std	4-F PhAc	–105.4	5.648

To evaluate whether relaxation delay settings could bias our quantitative results, qNMR experiments were performed using 4-fluorothiophenol and sulfone **1c** as model compounds with varying relaxation delays (d_1_ = 1, 10, 20, and 35 s). The resulting integrals for both analytes varied by less than ±5% in comparison to internal standard (4-fluoroacetophenone) (see SI for more information). Based on *T*_1_ values, we can conclude that species in higher oxidation states would exhibit even smaller deviations. These results indicate that d_1_ delay parameters of 1–5 s results in minimal quantitative error, with the greatest impact observed for sulfur-(II) species.

### Probing Disproportionation and Overreduction in Nickel Catalysis

The oxidation state-dependent fluoride shift could also be used to quickly identify and quantify various disproportionation byproducts commonly seen in reactions involving sulfinyl radicals. As the corresponding 4-fluorophenyl analog is known for each species (as shown in Figure 2), the detection and quantification by crude ^19^F NMR should be straightforward. To demonstrate this approach, two examples of nickel-catalyzed sulfonylation reactions from the literature were selected to look specifically for disproportionation or overreduction byproducts. We also utilized 4-fluorophenyl tagging to monitor byproduct formation in our own work on nickel-catalyzed coupling of NHMP sulfones.

In a first example, Rueping and co-workers utilized sodium sulfinate salts to synthesize aryl, heteroaryl, and vinyl sulfones from the corresponding halide using nickel/photoredox dual catalysis (Figure 4).^27^ While they obtained excellent yields (86%) with their model sulfinyl radical precursor, sodium 4-methylbenzenesulfinate (**5a**), yields diminished when using other sodium sulfinate salts. They reported a 54% yield with sodium 4-fluorobenzenesulfinate (**5b**). We believe this loss in product was most likely due to the formation of disproportionation byproducts. To demonstrate this, we analyzed a crude reaction mixture of **5b** by ^19^F NMR following Rueping’s conditions (Figure 4). In addition to the desired sulfur-(VI) product (**6b** δ −102.4 ppm), the sulfur-(II) sulfide (**7** δ −110.4 ppm) formed in an approximate 1:1 ratio.

**Figure 4:**
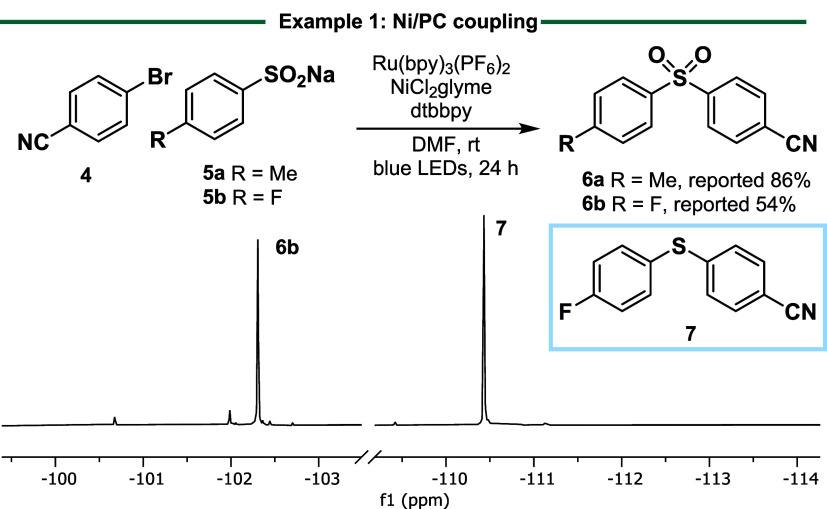
Ni/Photoredox dual catalysis for the synthesis of sulfones. Yield for **6a** and **6b** are reported by Rueping and co-workers.^27^ ^19^F NMR (400 mHz, CDCl_3_) of a repeated crude reaction mixture of **6b** with annotated assignments.

In a second example, Huang and co-workers utilized sulfinate salts as sulfenylating reagents to access new C–S bonds through visible light-induced nickel dual photocatalysis from aryl halides.^28^ The authors screened additives including the addition of water and monitored the conversion of 4-methylbenzene sulfinate (**5a**) to the sulfide product (**9a**) and unwanted sulfone byproduct (**10a**) by GC analysis with dodecane as an internal standard (Table 2). We repeated three conditions from their optimization table,^28^ replacing sodium 4-methylbenzenesulfinate (**5a**) with sodium 4-fluorobenzene sulfinate (**5b**). While yields obtained using the 4-fluorophenyl tag varied slightly from the reported GC values, general trends were maintained, providing a quick readout of product distribution. This reinforces the value of ^19^F NMR with the 4-fluorophenyl tag as a viable alternative to GC/MS for reaction optimization. This prompted us to next use the 4-fluorophenyl tag to monitor product formation in our own efforts toward the synthesis of complex sulfur-(VI)-containing small molecules from NHMP sulfones.

**Table 2:**
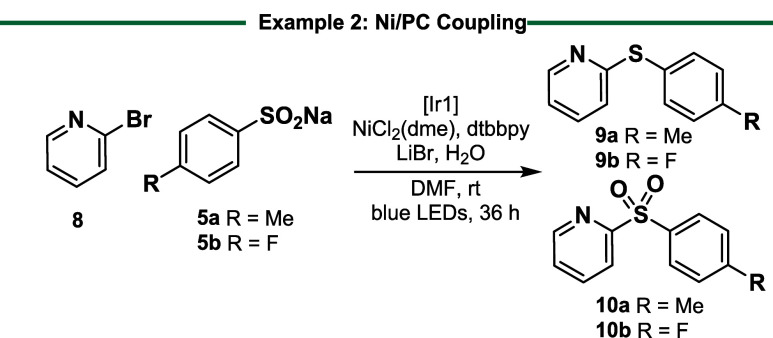
Deoxygenative C–S Bond Coupling

	R = Me – GC yield^a^		R = F – ^19^F yield^b^	
conditions	**9a**	**10a**	**9b**	**10b**
standard	92%	8%	93%	7%
5 equiv water	26%	25%	53%	21%
NaCl instead of LiBr	57%	15%	66%	n.d.

a Literature conditions and reported yields via GC analysis with dodecane as an internal standard from ref 27.^27^

b Our yields reported from ^19^F NMR tagging with 4-fluoroacetophenone as an internal standard. [Ir1] = [Ir­(dF­(CF_3_)­ppy)_2_(dtbbpy)]­PF_6_.

In our efforts toward the coupling of NHMP sulfone precursors (**1c**), we attempted a nonphotocatalytic cross-coupling with aryl halides with the goal of obtaining aryl sulfones. Given that Weix^29^ showed that *N*-hydroxyphthalide esters could couple to aryl iodides under nickel catalysis, we assumed that similar reactivity could yield aryl sulfones (**12**) from the NHMP sulfone precursor (**1c**) (Figure 5). Upon reaction of **1c** with 4-iodotoluene in the presence of Zn^0^ and NiBr_2_(dtbbpy), ^19^F shifts in the sulfur-(II) range were observed in crude NMRs, which were confirmed to be the disulfide (**14** δ −113.4 ppm) and sulfide (**13** δ −115.0 ppm) disproportionation byproducts (Figure 5, entry 1). No evidence of the sulfone **12** (expected at ca. δ −102.5 ppm) was observed. Increasing the temperature to 60 °C increased formation of the sulfide byproduct **13** and eliminated the disulfide byproduct **14** (Figure 5, entry 2). Changing the solvent from DMA to DMSO resulted in formation of the disulfide, with the NHMP starting material remaining (δ −101.7 ppm) (entry 3). Finally, switching the ligand from dtbbpy to Bphen resulted in increased amounts of NHMP starting material, with minimal formation of the observed byproducts (entry 4). After exhaustive solvent and ligand screens, we concluded that these conditions would consistently result in the over-reduction of our intended sulfur-(VI) product affording the sulfur-(II) byproducts exclusively. We transitioned to photocatalytic methods that ultimately led to our published work for the sulfonylation of anilines.^16^

**Figure 5:**
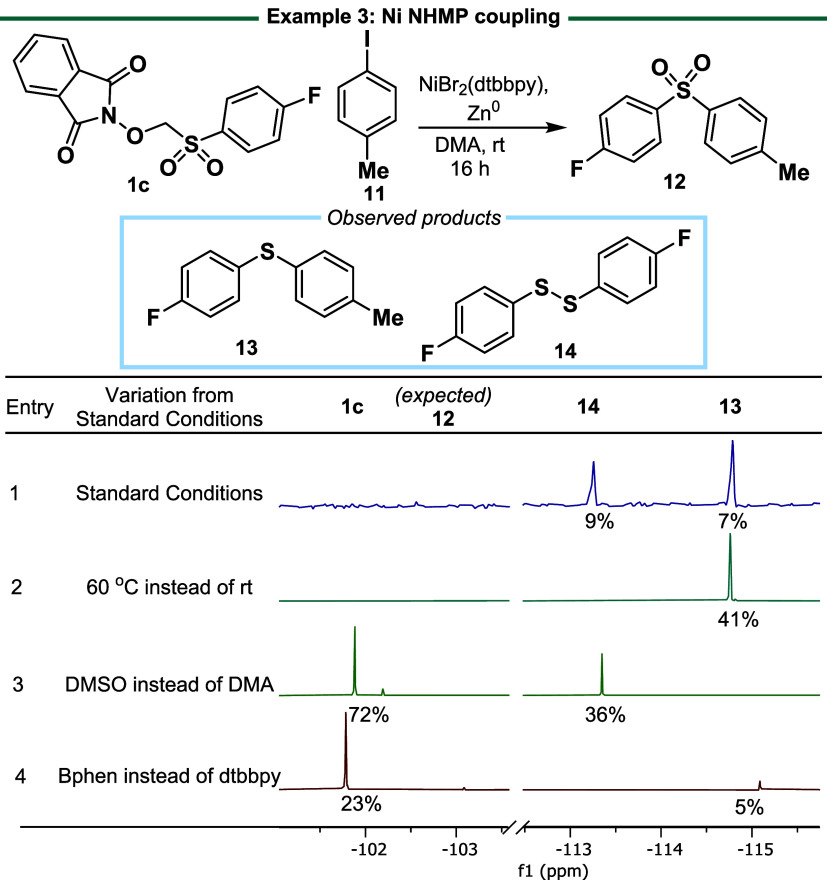
Representative examples of our efforts in the nonphotocatalytic cross-coupling of aryl halides with NHMP reagent **1c**, with 4-fluoroacetophenone added as an internal standard, ^19^F NMR (400 mHz, CDCl_3_). The area between −103.5 ppm and −112.5 ppm contained only the 4-fluoroacetophone peak and was omitted for clarity. Yields shown are NMR yields using 4-fluoroacetophenone as the internal standard. See SI for full spectra; (a) Standard conditions: 4-iodotuluene (1 equiv), **1c** (1.5 equiv), NiBr_2_(dtbbpy) (10 mol %), Zn^0^ (2 equiv), DMA (0.4 M), rt, 16 h. Bphen = Bathophenanthroline.

### Probing Disproportionation in Photocatalytic Aniline Sulfonylation

In these recent studies toward the sulfonylation of aniline substructures using the NHMP sulfone as a sulfinyl radical precursor, we initially selected *fac*-Ir­(ppy)_3_ as the photocatalyst. The excited state photocatalyst would reduce the NHMP sulfone to yield the sulfinyl radical. With these conditions, reproducibility was problematic with high sensitivity to oxygen and water leading to increased amounts of disproportionation byproducts. These byproducts were readily characterized using ^19^F NMR and the 4-fluorophenyl tag. As the oxidation state of sulfur byproducts in the reaction mixture can be characterized by ^19^F NMR shifts alone, initial peak assignments could be made (Figure 6a): δ −113 ppm is related to sulfide products, δ −104 ppm to −106 ppm contains a sulfone product, and the desired sulfonylation product **16** was found closer to δ −106 ppm. It was difficult to obtain a reliable standard NMR of the 4-fluorobenzenesulfonic acid (**20**) in CDCl_3_, but in D_2_O and MeOH-d4, the shift is generally from δ −109 ppm to −113 ppm.^30,31^ These initial assignments were later confirmed by ^1^H NMR after isolation. A list of all 4-fluorophenyl disproportionation products observed and isolated during the scope of this optimization with their ^19^F chemical shifts can be seen in Figure 6b. In this case, ^19^F NMR of crude reaction mixtures with 4-fluoroacetophenone added as an internal standard allowed for fast and efficient identification and quantification of byproducts and yields thereby streamlining our optimization efforts.

**Figure 6:**
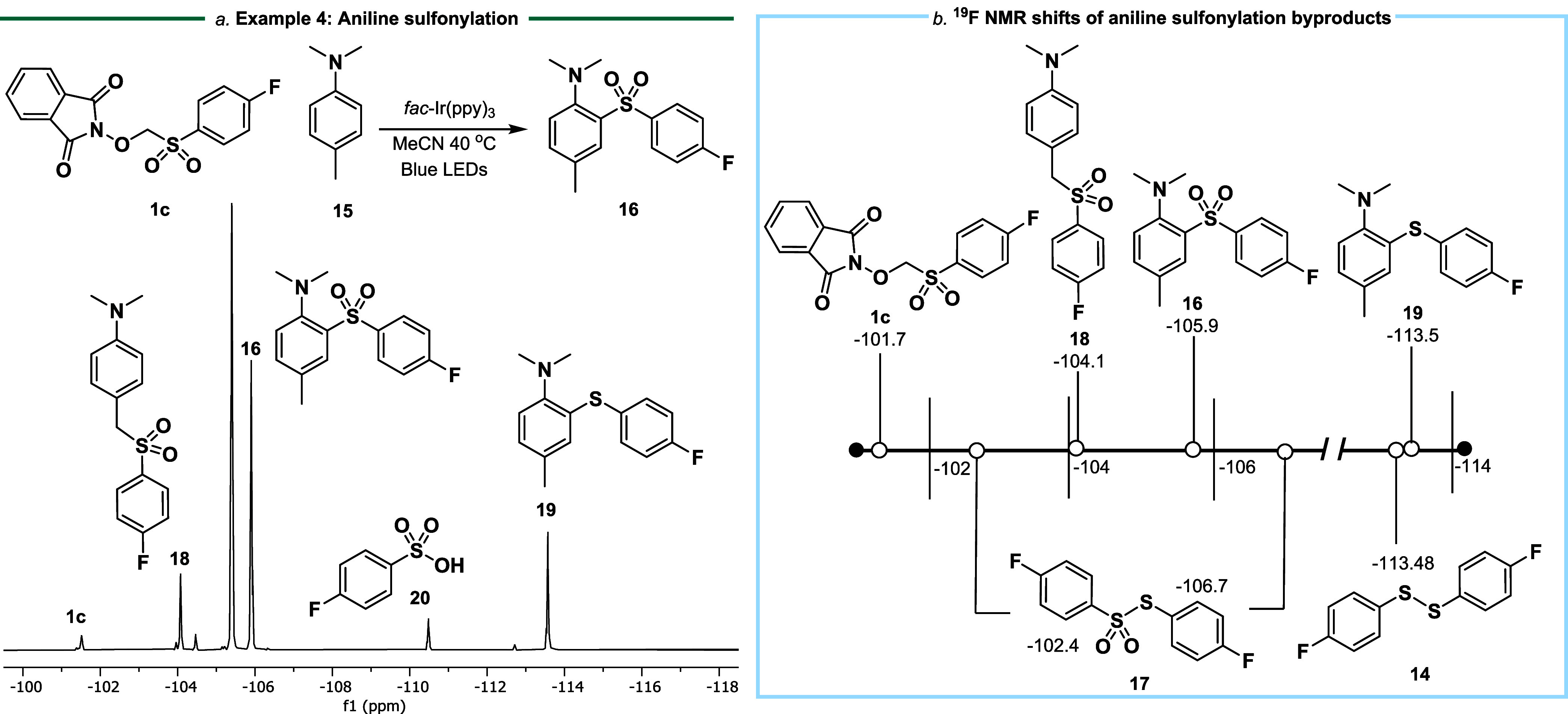
a. Crude reaction mixture of initial aniline sulfonylation efforts. Conditions: **1c** (2 equiv), **15** (1 equiv), *fa*c-Ir­(ppy)_3_ (2 mol %), MeCN (0.1 M), blue LEDs, 16 h. 4-fluoroacetophenone added as an internal standard and referenced to δ −105.4 ppm; b. All products and byproducts observed over the course of all aniline sulfonylation optimizations. Shifts shown in ppm.

### Monitoring Reaction Progress

To further demonstrate the utility of ^19^F NMR as a powerful, high-throughput tool for monitoring reaction yields during optimization campaigns, we applied this approach to the EDA-mediated hydrosulfonylation of olefins using sulfonyl radical precursor **1c**, as reported in our recent publication.^17^ While the reported optimization focused on the yield of product **21**, the simultaneous observation of byproducts **22** and **23**, as well as unreacted starting material **1c**, provided valuable insight into the performance of each reaction condition (Table 3).

**Table 3:**
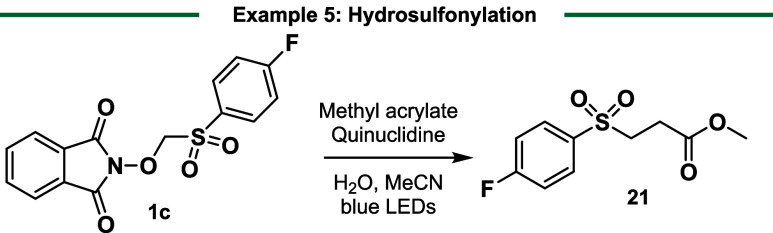
Hydrosulfonylation Optimization

	NMR yields (%)^a^			
	**1c**	**21** ^b^	**22**	**23**
deviation from standard conditions	δ −101.7	δ −102.9	δ −112.0	δ −113.1
**standard**		96	7	
aceclidine	23	4	6	
Et_3_N	27	30	11	
DABCO	16	74	21	
no blue LEDs	2	84	6	5
purple LEDs	11	17	51	

a Conditions unless otherwise noted: sulfone **1c** (1 equiv), methyl acrylate (5 equiv), quinuclidine (1 equiv), degassed MeCN (0.1 M), H_2_O (10 equiv), blue LEDs (440 nm), 16 h; ^19^F NMR yield with 4-fluoroacetophenone as an internal standard.

b NMR yields for **21** repeated from our previous publication.

The utility of this approach can be further extended through real time reaction monitoring by acquiring ^19^F NMR spectra at defined time intervals to generate reaction progress curves (Figure 7). This can be obtained either by the addition of a standard to the reaction or by removing aliquots and adding an internal standard. While this study uses the latter method with 4-fluoroacetophenone as the internal standard, other options are functional here, such as trifluorotoluene. To demonstrate this capability, the formation of product **21** using standard conditions was monitored over the course of 2 h. A steady increase in the concentration of **21** was observed. An initial increase in **1c** was also detected as the starting material gradually dissolved in acetonitrile, followed by a decrease as it was consumed during the reaction. In addition to product formation, species **22**, **23**, and **24** were observed throughout the reaction. Analysis of the reaction progress curve suggests that **23** and **24** may be reaction intermediates, as their concentrations increase during the first hour before declining as product **21** forms. In contrast, **22** appears to be a byproduct, as it accumulates throughout the course of the reaction. Although compounds **22**, **23**, and **24** could not be isolated and definitively characterized, their ^19^F NMR chemical shifts provide insight into their likely oxidation states. Based on these shifts, **24** is proposed to contain a sulfur-(VI) functionality, whereas **22** and **23** are more consistent with sulfur-(II) species. The ability to rapidly identify and quantify multiple fluorinated species from a single spectrum highlights the utility of ^19^F NMR as a high-throughput platform for reaction optimization, reaction monitoring, and mechanistic investigation.

**Figure 7:**
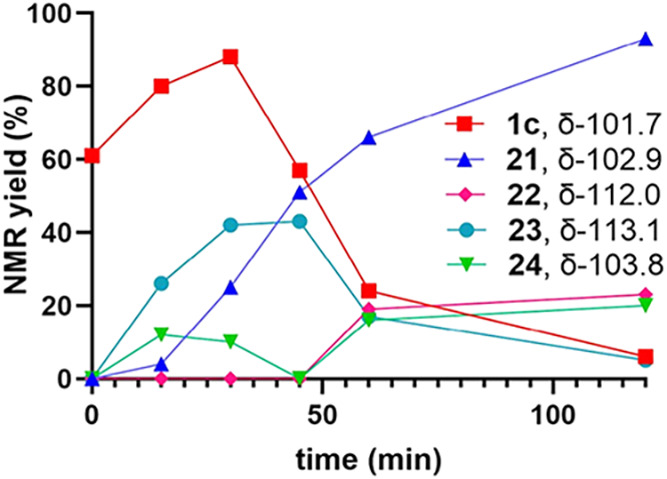
Reaction progress curve of hydrosulfonylation (example 5) under standard conditions.

We envision the ^19^F phenyl-tagging as a method to streamline optimization of sulfur-based reactions and scope studies providing clean, interpretable spectra without the need for isolation of each byproduct. Overall, when compared to additional methods such as GC/MS or LCMS, the 4-F phenyl tagging can act as a complementary method that can provide additional data to aid in reaction optimization.

## Conclusion

In conclusion, adding a 4-fluorophenyl tag to sulfur-containing molecules allows for rapid characterization of products and byproducts in sulfur-based transformations via ^19^F NMR. This can be a useful tool for mechanistic analysis and product profiling in diverse areas of sulfur chemistry, as other conventional spectroscopic methods often cannot easily differentiate between sulfur oxidation states or monitor interconversion between these redox states during reaction progression. We anticipate that the data compiled in Figure 2 will be valuable to a wide range of organosulfur redox chemistry.

## Supplementary Material



## Data Availability

The data underlying this study are available in the published article and its Supporting Information.

## Notes

Experimental procedures, compound characterization, and supporting figures (PDF)
