# Supplementary material for: 4‑Fluorophenyl Tagging as a Diagnostic Tool for Monitoring Sulfur Oxidation States via 19F NMR
Source: J Org Chem. 2026 Jul 22;91(30):10446–53. doi: 10.1021/acs.joc.6c00917 (PMC13435314; doi:10.1021/acs.joc.6c00917)
Supplement: Supplementary file 1 [file jo6c00917_si_001.pdf]

Supporting Information

4-Fluorophenyl Tagging as a Diagnostic Tool for Monitoring Sulfur Oxidation States via ^{19}F NMR

Makayla L. Williams, Twinkle I. Patel, and Matthew J. Moschitto*

Department of Medicinal Chemistry, Rutgers, The State University of New Jersey, Piscataway, NJ 08854-8020, USA

Table of Contents

General Information 3
Selection of Internal Standards 3
Known ^{19}F Chemical Shifts of 4-fluorophenyl containing molecules 4
Oxidation Monitoring of NHMP Reagent 6
LOD/LOQ 6
T1 Relaxation Calculations 7
Use of 4-Fluoro tagging in Literature Conditions 10
Use of 4-Fluoro tagging in non-photocatalytic cross-coupling efforts 10
Use of 4-Fluoro tagging in Aniline Sulfonylation 12
Real-time reaction monitoring 14
Characterization and Spectra of Isolated Compounds 15
References 19

General Information

All chemicals were purchased from Ambeed, Sigma Aldrich, or ThermoFisher Scientific and used without purification. ACS grade MeCN was purchased from Fisher Chemicals and used without purification. Yields refer to chromatographically and spectroscopically (^{1}H NMR) homogeneous material unless otherwise noted. Analytical thin-layer chromatography (TLC) was performed using Merck Silica Gel 60 Å F-254 precoated plates (0.25 mm thickness), and components were visualized by ultraviolet light (254 nm) and/or by staining with $KMnO_4$ or phosphomolybdic acid. Flash column chromatography was performed on a Teledyne CombiFlash NextGen 300 automated flash purification system with various Teledyne cartridges (4-80 g, 40-63 µm, 60 Å). Purifications were performed with hexanes and ethyl acetate unless otherwise noted.

High-resolution mass spectral data were obtained on a Waters SYNAPT XS (TOF) high resolution mass spectrometer using electron spray ionization (ESI) in the positive ion mode. All photoredox reactions were performed using a Kessil PR160L-Blue LED lamp (30 W, λmax = 440 nm, 100% intensity, 5 cm from the wall of vial).

^{1}H and ^{13}C NMR spectra were recorded on a Bruker Advance-III spectrometer with a BBO probe at 400 MHz and 101 MHz, respectively, in $CDCl_3$, MeOD, or DMSO-*d*6. Chemical shifts were reported in ppm; multiplicities are indicated by s = singlet, d = doublet, t = triplet, q = quartet, p = pentet, sep = septet, dd = doublet of doublet, dt = doublet of triplet, dp = doublet of pentet, m = multiplet, br = broad resonance. Coupling constants '*J*' were reported in Hz. All ^{19}F NMR experiments were run with proton decoupling with a d_1 delay of 1 s unless otherwise noted.

Selection of Internal Standards

Throughout the scope of this manuscript, 4-fluoroacetophenone was employed as the internal standard as its ^{19}F NMR peak is well resolved from those of the analytes and the compound is nonvolatile. Other fluorinated compounds, such as trifluorotoluene, may also be suitable. However, internal standards containing acidic protons should be avoided to minimize the possibility of undesired interactions with species present in the reaction mixture.

Known ^{19}F Chemical Shifts of 4-fluorophenyl containing molecules

Table S1: ^{19}F Chemical Shifts of 4-fluorophenyl containing molecules

Structure	^{19}F Shift	Reference
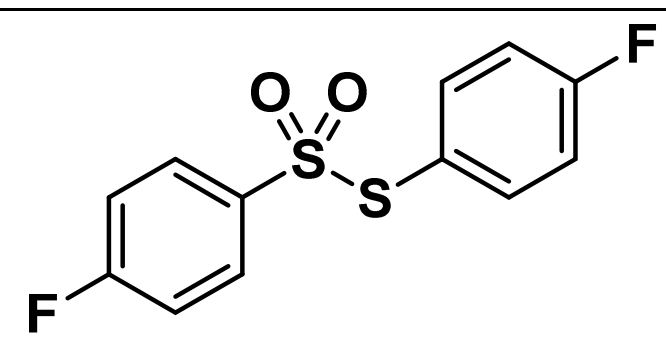	-102.42<br>-106.77	[1]
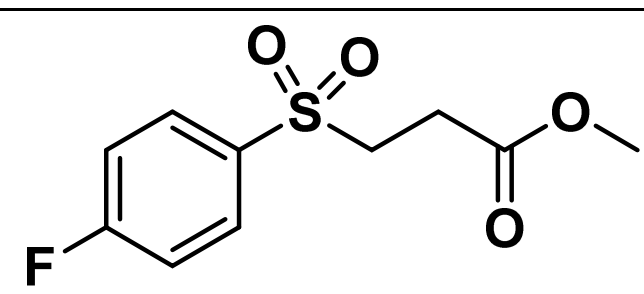	-102.7	[2]
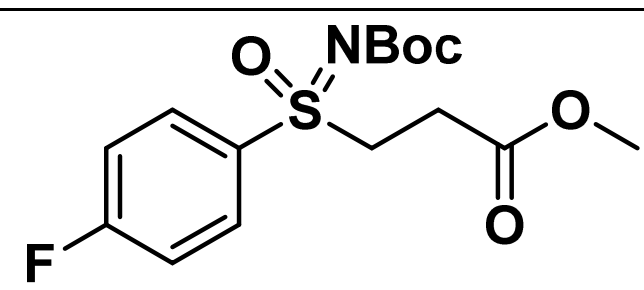	-103.0	[3]
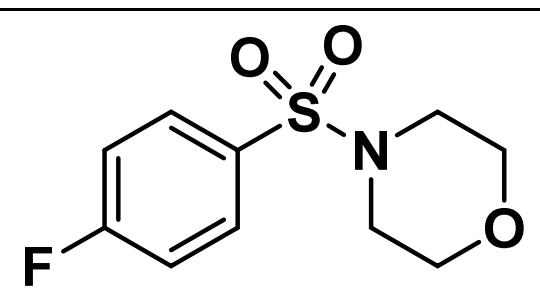	-104.51	[4]
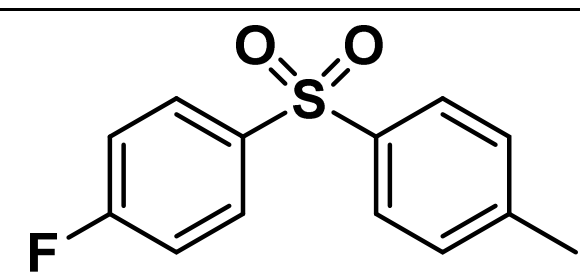	-104.6	[5]
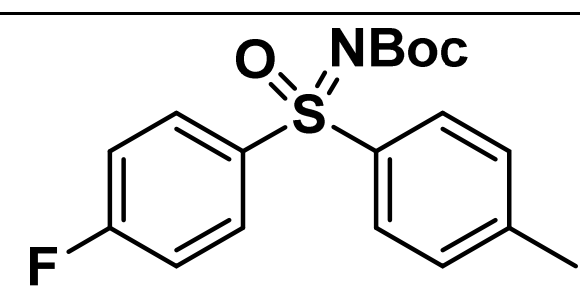	-104.71	[6]
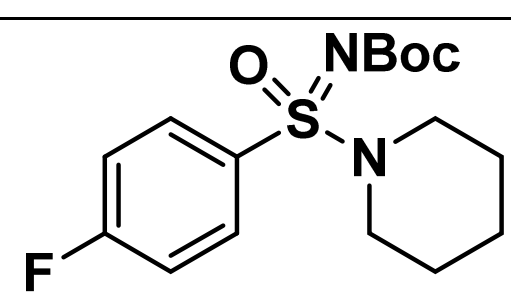	-105.2	[3]
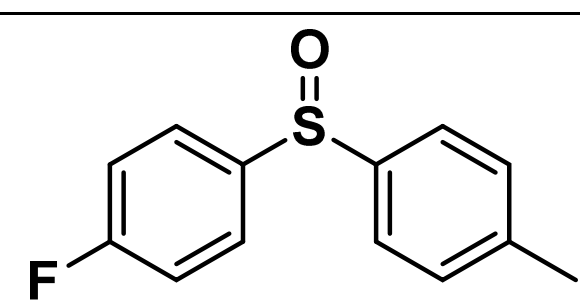	-106.66	[7]
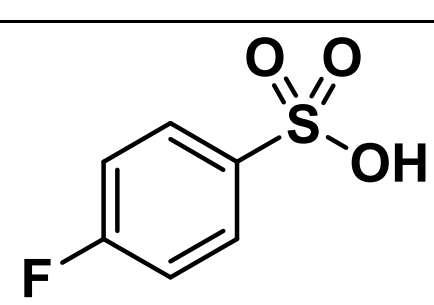	-107.0	[8]

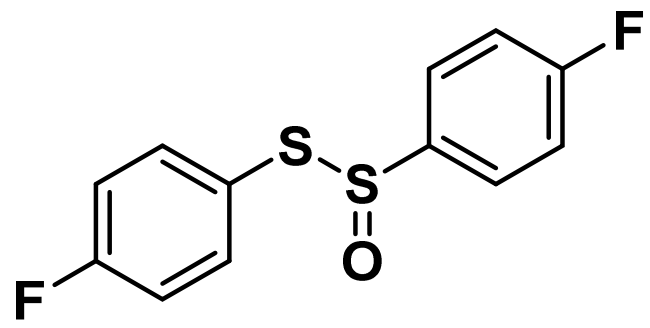	-107.1<br>-109.1	[9]
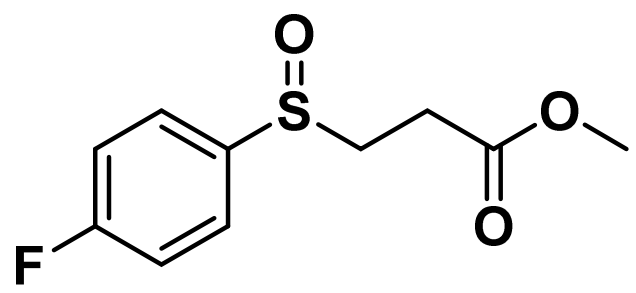	-108.3	[3]
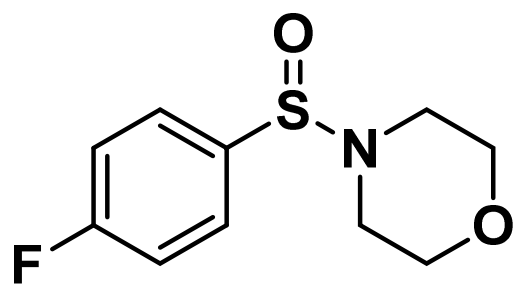	-108.9	[10]
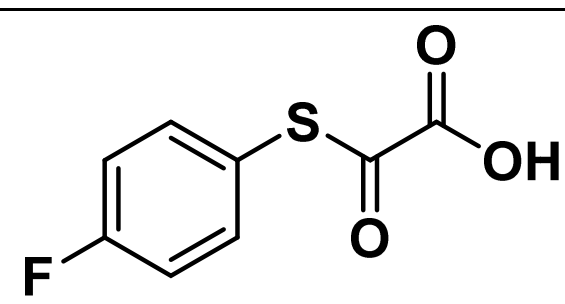	-109.0	[11]
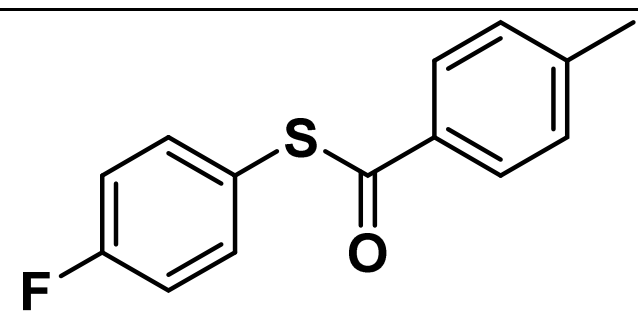	-111.2	[11]
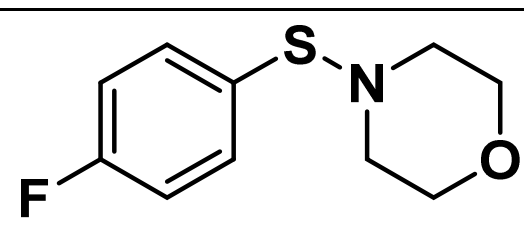	-112.2	[12]
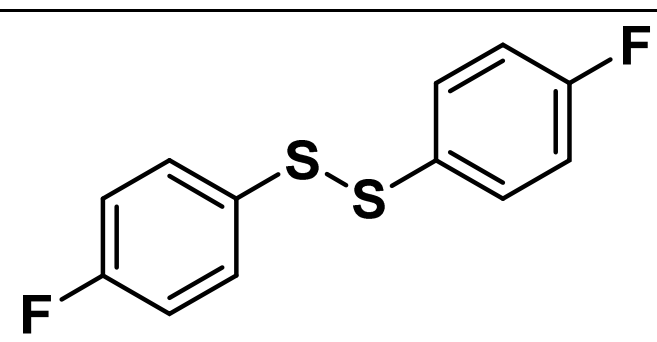	-113.42	[1]
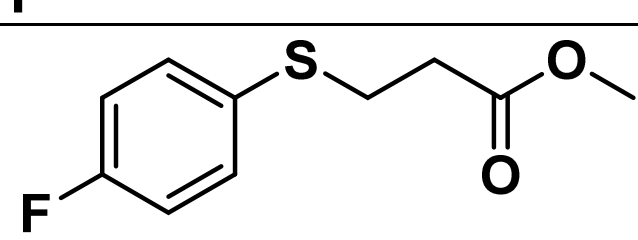	-114.6	[3]
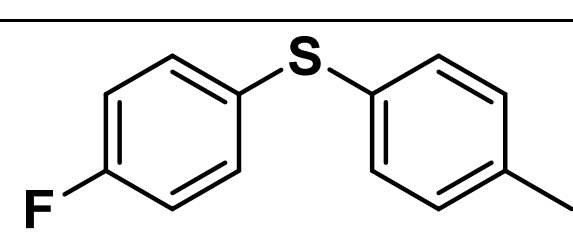	-115.06	[13]

Oxidation Monitoring of NHMP Reagent

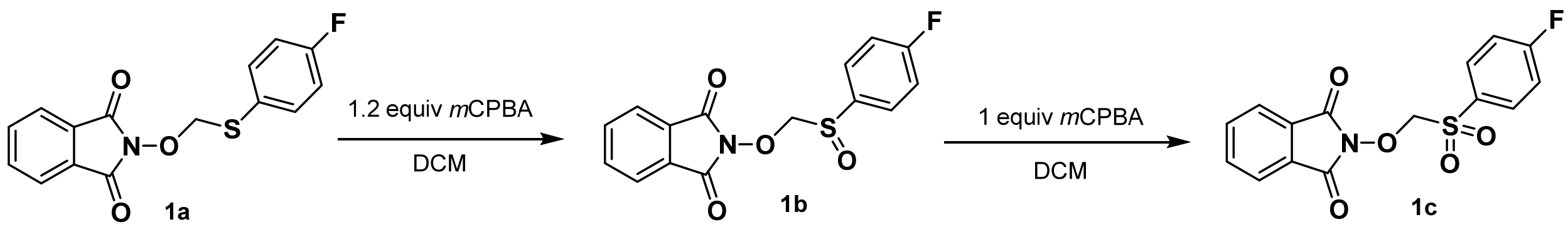

1a was synthesized according to the previously reported literature procedure using paraformaldehyde, conc HCl, and toluene from 4-fluorobenzene thiol. [14] To a stirred solution of **1a** (10g, 32.97 mmol) in CH_2Cl_2 (370 mL, 0.089 M), *m*CPBA (1.2 equiv, 75% in water) was slowly added at 0°C. The resulting solution was stirred for 4 h at room temperature. Crude ^{19}F NMR of the reaction mixture was taken at this point to monitor conversion to the sulfoxide **1b** (with small amounts of sulfone present). Addition of another equivalent of *m*CPBA (1 equiv, 75% in water) at 0°C stirred for 4 h at room temperature resulted in full conversion to the sulfone. The reaction mixture was concentrated under reduced pressure. Diethyl ether was added to the resulting residue and the precipitates were filtered and washed with diethyl ether (x3) to obtain the desired product **1c** as a white solid (10.7 g, 97% yield). The spectral data matched with that of literature. [14]

LOD/LOQ

The limit of detection (LOD) and limit of quantification (LOQ) are important validation parameters for analyte identification and quantification. The LOD can be determined either mathematically or visually from an NMR spectrum and is commonly defined as the concentration corresponding to a signal-to-noise ratio (SNR) of 3. [19] For quantitative measurements with a relative uncertainty of 1%, a minimum SNR of 150 is generally required to achieve the LOQ.[19] Using our probe to acquire decoupled ^{19}F NMR spectra with 16 scans, we can reliably detect signals corresponding to concentrations as low as 0.4 mM and accurately quantify analytes at concentrations of approximately 4 mM or greater. Both the LOD and LOQ can be further improved by increasing the number of scans. These parameters are also influenced by several experimental factors, including the operating frequency of the instrument, acquisition parameters, sample conditions, and the post-processing parameters applied during spectral analysis. Consequently, the LOD and LOQ reported here are specific to the experimental conditions used in this study and may differ under other instrument or acquisition conditions.

A 1 M solution of 4-fluoroacetophenone was prepared by dissolving 121.4 µL in 878.6 µL of $CDCl_3$. A 10 mM stock solution was prepared by diluting 5 µL of the 1 M solution with 495 µL of $CDCl_3$.
A 5 mM solution was prepared by taking 250 µL of the 10 mM stock and diluting with 250 µL $CDCl_3$.
A 2 mM solution was prepared by taking 100 µL of the 10 mM stock and diluting with 400 µL $CDCl_3$.
A 1.2 mM solution was prepared by taking 60 µL of the 10 mM stock and diluting with 440 µL $CDCl_3$.
A 0.6 mM solution was prepared by taking 30 µL of the 10 mM stock and diluting with 470 µL $CDCl_3$.

These solutions were then transferred directly to an NMR tube. Decoupled ^{19}F NMR was obtained with 16 scans. All spectra were processed using sine-squared apodization with a 90° phase shift, followed by automatic phase correction and baseline correction. Mestrenova's SNR Calculator was then used to

determine the SNR of the resulting peak in each solution. The resulting SNR values were then plotted against concentration, and a linear regression was applied to establish the relationship between analyte concentration and SNR.[19]

^{19}F{^{1}H} NMR ($CDCl_3$, 376 MHz)

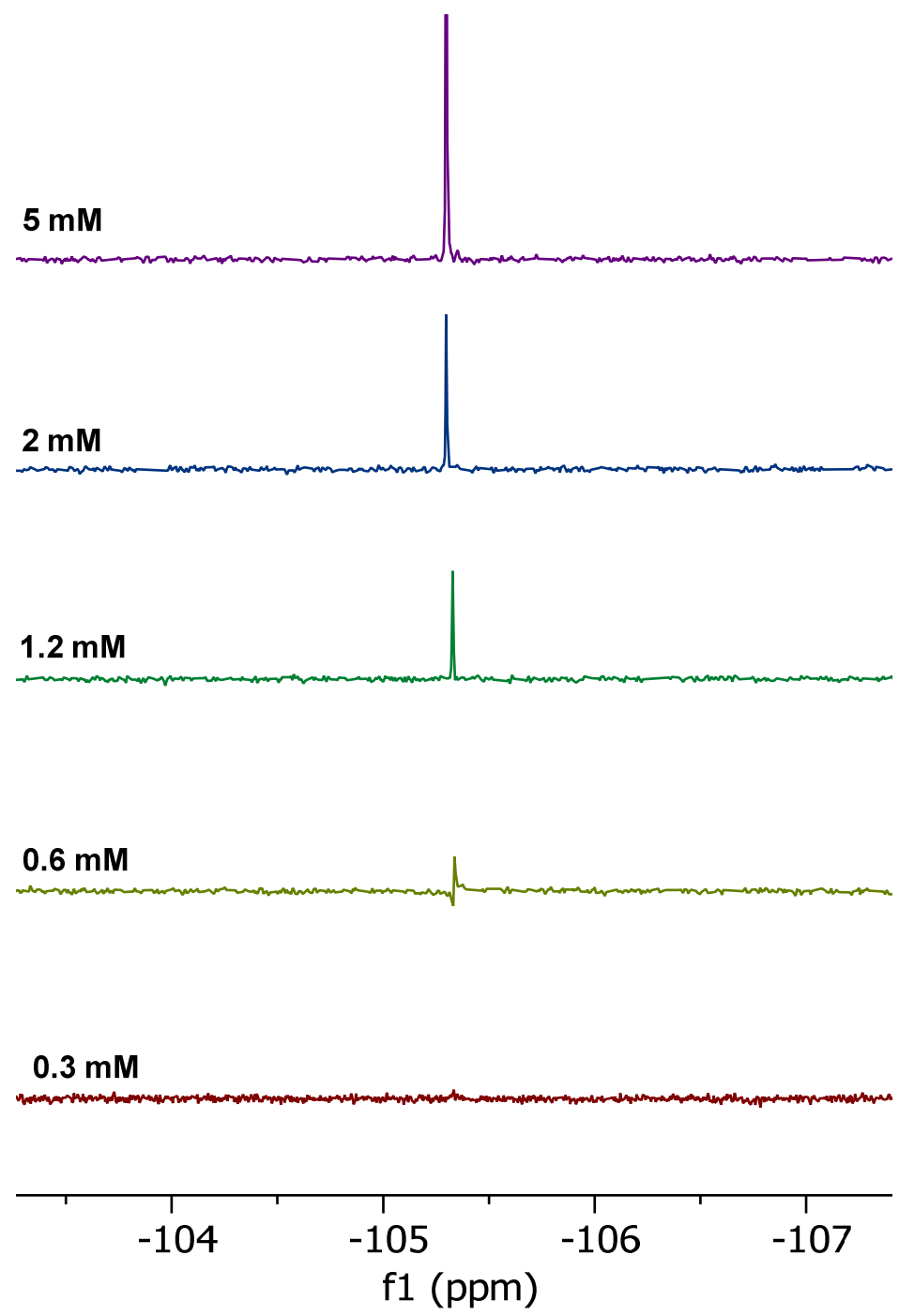

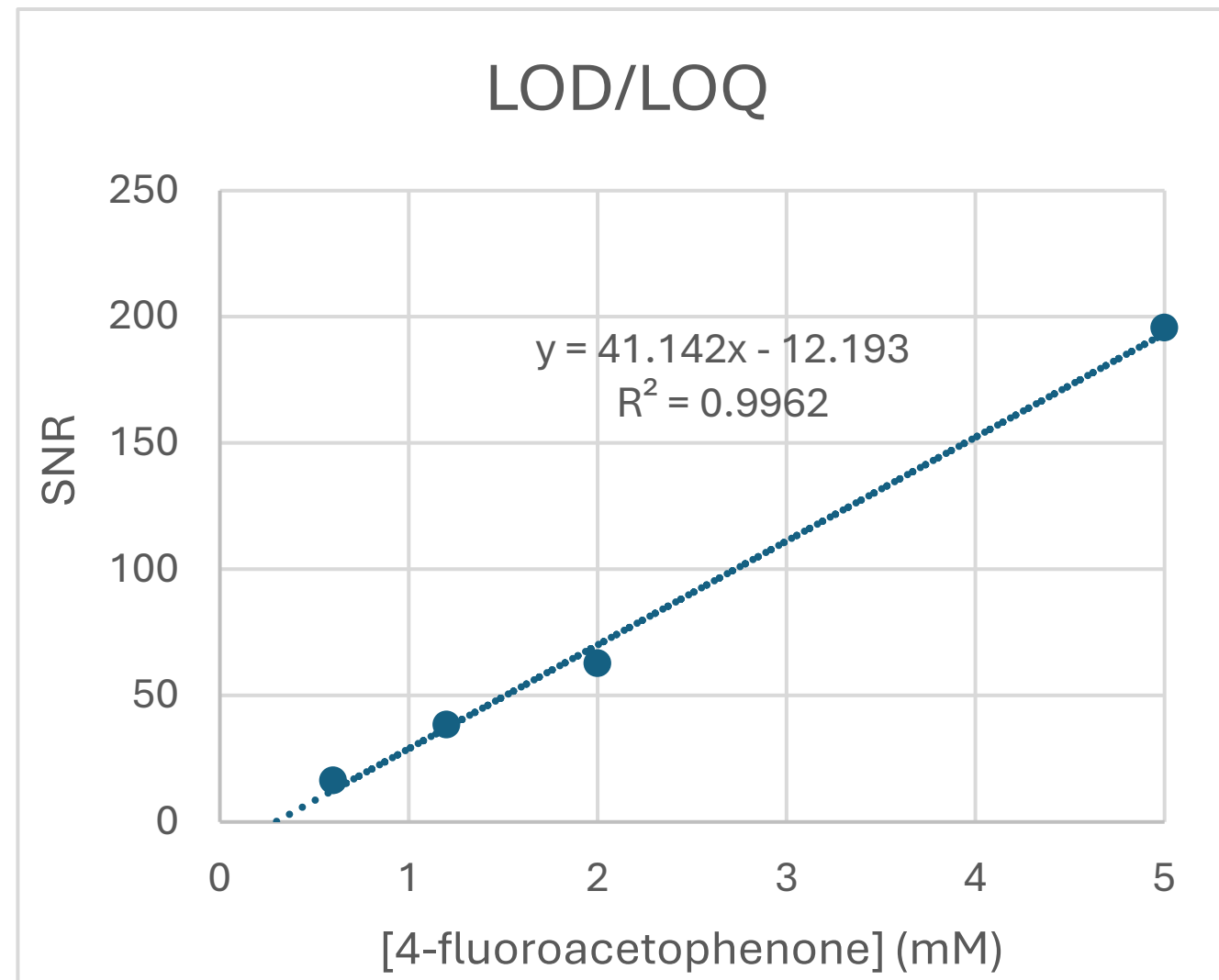

LOD: SNR of 3 = 0.38 mM

LOQ: SNR of 150 = 3.94 mM

Figure S1: Left: 19F NMR of 4-fluoroacetophenone at varying concentrations (ns=16); right: plot of SNR and concentration to determine LOD and LOQ.

T1 Relaxation Calculations

In quantitative NMR (qNMR), accurate signal integration requires consideration of the longitudinal relaxation time (T_1), which varies among nuclei and is influenced by factors such as solvent, pH, salt concentration, oxygen content, and magnetic field strength. To ensure reliable quantification, the repetition time (relaxation delay plus acquisition time) should be at least five to seven times the longest T_1 value of the signals being analyzed, corresponding to approximately 99.0–99.9% signal recovery.[19] Insufficient relaxation delays can lead to incomplete spin relaxation between scans, resulting in systematic integration errors and inaccurate concentration measurements. Given that the default relaxation delay on our NMR instrument is 1 s (d_1), we evaluated whether this setting could compromise quantitative accuracy.

To assess the impact of longitudinal relaxation on quantitative accuracy, we measured T_1 values for a representative set of eight molecules in our system. These measurements revealed that sulfur-(II) species exhibit longer T_1 relaxation times compared to sulfur-(IV) and sulfur-(VI) analogues.

To evaluate whether relaxation delay settings could bias our quantitative results, qNMR experiments were performed using 4-fluorothiophenol as a model compound with varying relaxation delays (d_1 = 1, 10, 20, and 35 s). The resulting integrals indicated that 4-fluorothiophenol varied by less than ±5% in comparison to internal standard (4-fluoroacetophenone)(see SI for more information). We can conclude based on T_1 values that species in higher oxidation states would exhibit even smaller deviations. These results indicate that, under the acquisition parameters used in this study, incomplete relaxation introduces only minor quantitative error, with the greatest impact observed for sulfur-(II) species.

Table S2: T1 values for various sulfur species

Structure	Shift (ppm)	T_1
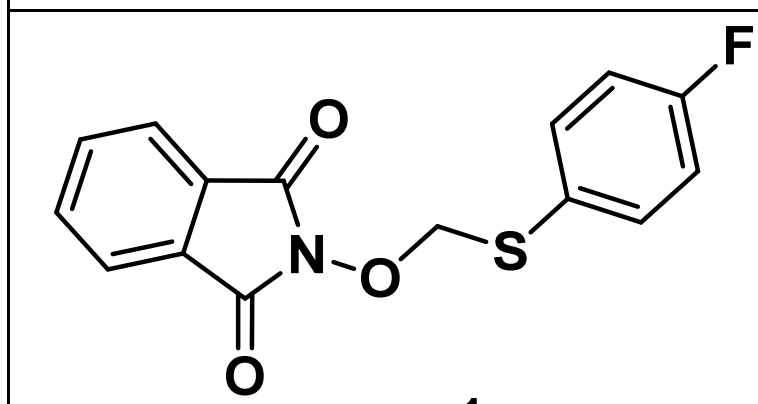 1a	δ -113.3	3.920 s
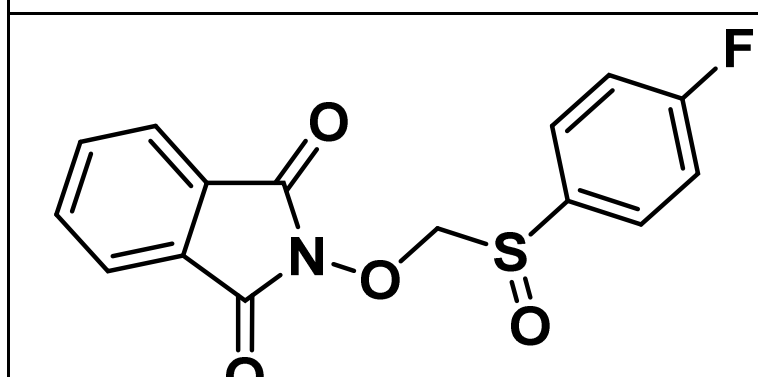 1b	δ -107.0	2.364 s
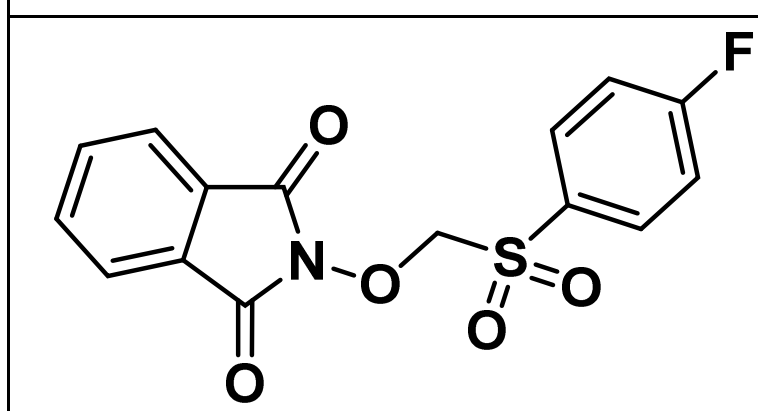 1c	δ -101.7	2.357 s
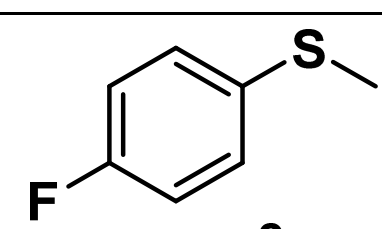 2a	δ -117.4	8.279 s
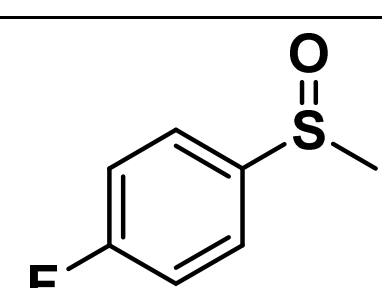 2b	δ -108.3	5.573 s
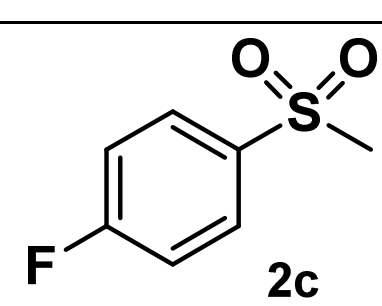 2c	-103.6	5.291 s
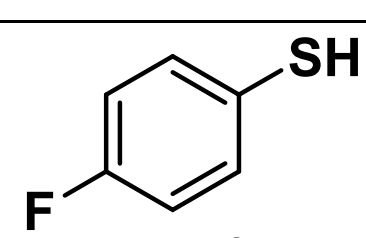 3	δ -116.6	5.865 s

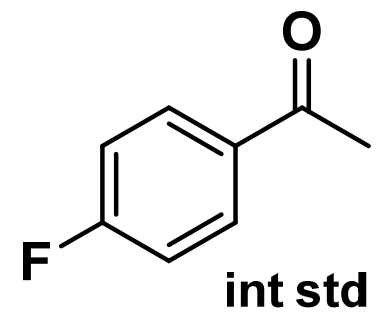 **int std**	δ -105.4	5.648 s

Samples were prepared directly in an NMR tube with $CDCl_3$. T_1 times were calculated by using the standard t1ir pulse program in TOPSPIN.

Raw data can be found in the table below.

Table S3: Raw integral data for calculation of T1 values

					Integral (x10^9)			
**tau**	**1c**	**1b**	**1a**	**2a**	**2b**	**2c**	**3**	**int std**
0.01	1.62E+00	2.8508	3.3348	1.3979	2.2815	8.87E-01	1.2833	2.4211
0.05	1.6443	2.8375	3.3171	1.3878	2.2856	8.95E-01	1.2801	2.4048
0.1	1.6432	2.8173	3.2933	1.3894	2.289	8.93E-01	1.2776	2.4194
0.25	1.6572	2.8633	3.3286	1.398	2.2875	8.94E-01	1.2838	2.4384
0.5	1.6619	2.9165	3.3741	1.4077	2.3072	9.02E-01	1.2854	2.446
1	1.6893	2.9631	3.4495	1.4279	2.3336	9.09E-01	1.3094	2.4738
2	1.7365	3.0692	3.5234	1.4539	2.405	9.21E-01	1.3372	2.5188
3	1.7236	3.1013	3.6034	1.4965	2.4532	9.46E-01	1.3382	2.5506
4	1.7666	3.1249	3.6844	1.5388	2.4772	9.51E-01	1.4407	2.5905
8	1.7851	3.213	3.7996	1.6093	2.5842	9.80E-01	1.4766	2.6752
**T1**	2.357 s	2.364 s	3.92s	8.279 s	5.573 s	5.291 s	5.865 s	5.648 s

To evaluate how relaxation delay settings vary quantitative results, qNMR experiments were performed using a mixture of 4-fluoroacetophenone (**int std**) with 4-fluorothiophenol (**3**) and NHMP sulfone **1c** as model compounds, with varying relaxation delays (d_1 = 1, 10, 20, and 35 s). Integral ratios were obtained as indicated below.

Table S4: Ratios of standard to compounds at varying d_1 values

d_1	Int std:3	Int std:1c
1 s	1:0.94	1:0.53
10 s	1:0.90	1:0.50
20 s	1:0.90	1:0.50
35 s	1:0.90	1:0.50

This indicates that experiments conducted using the default 1 s relaxation delay may deviate in quantitative yield by up to 4.44%. The data also suggests that the measured ratio reaches a plateau at a relaxation delay of approximately 10 s, indicating that extending the delay beyond this value (d_1 > 10 s) provides minimal additional improvement in quantitative accuracy under the conditions studied.

Use of 4-Fluoro tagging in Literature Conditions

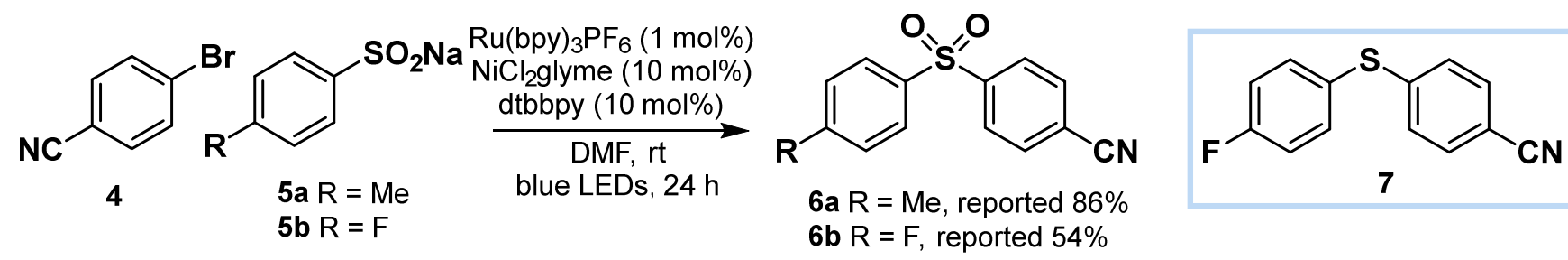

Following literature procedure, [15] a sealed vial charged with 4-bromobenzonitrile (18.2 mg, 0.1 mmol, 1 equiv), sodium 4-fluorobenzenesulfinate (36.4 mg, 0.2 mmol, 2 equiv), Ru(bpy)$_3$PF$_6$ (0.5 mol%, 0.4 mg)*, NiCl$_2$·glyme (2.2 mg, 0.01 mmol, 10 mol%) and dtbbpy (2.7 mg, 0.01 mmol, 10 mol%) was evacuated and backfilled with N_2 (x3). Degassed DMF (1 mL, 0.1 M) was added to the vial via syringe. The reaction was irradiated at 40°C for 24 h at approximately 5.0 cm from a Kessil blue LED (λ_{max}= 440 nm, 100% intensity). Upon completion, 500 µL of the reaction mixture was transferred directly to a NMR tube and diluted with $CDCl_3$. ^{19}F NMR of the crude reaction mixture was analyzed to identify formation of both the sulfone (**6b**) and sulfide (**7**). Spectral data matched with that of literature. [15, 16]

*Note: The paper by Rueping and others uses [Ir(tbppy)$_2$(dtbbpy)]PF$_6$ as their optimal photocatalyst. Due to commercial availability, Ru(bpy)$_3$PF$_6$ was selected from their optimization table as a viable alternative.

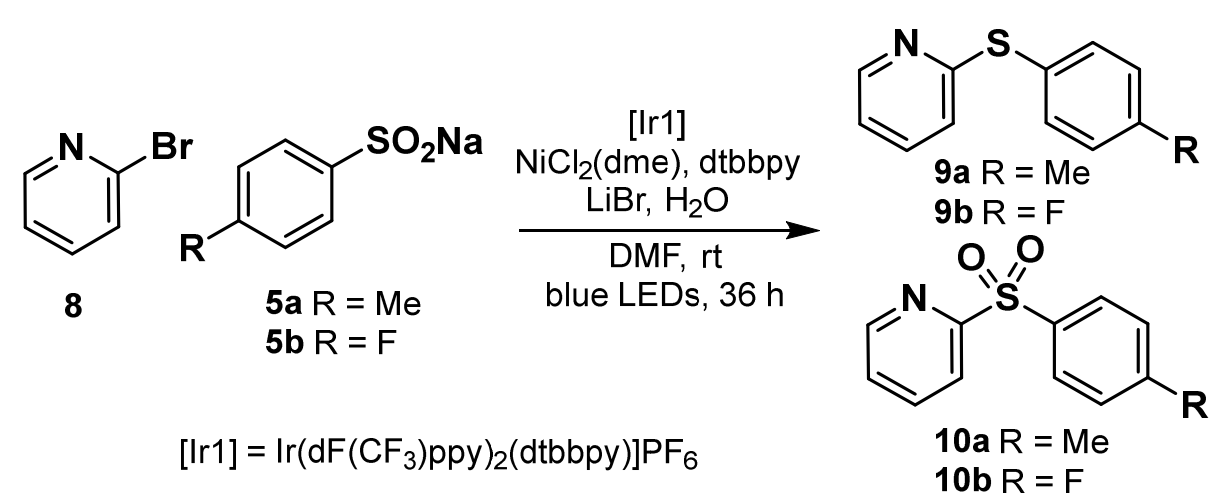

Following literature procedure, [17] a sealed vial charged with LiBr (4.1 mg, 0.474 mmol, 0.5 equiv), sodium 4-fluorobenzenesulfinate (34.6 mg, 0.1898 mmol, 2 equiv), [Ir(dFCF$_3$ppy)$_2$(dtbpy)]PF$_6$ (2.1 mg, 2 mol%), NiCl$_2$·glyme (1 mg, 5 mol%), and dtbbpy (1.3 mg, 5 mol%) was evacuated and backfilled with N_2 (x3). DMF (0.5 mL, 0.2 M), water (0.4 µL, 0.25 equiv), and 2-bromopyridine (9 µL, .0949 mmol, 1 equiv) were then added via syringe. The resulting mixture was then stirred at RT for 36 h at approximately 5.0 cm from a Kessil blue LED (λ_{max}= 440 nm, 100% intensity). Upon completion, 500 µL of the reaction mixture was transferred directly to a NMR tube and diluted with $CDCl_3$. ^{19}F NMR of the crude reaction mixture was analyzed to identify formation of both the sulfide (**9b**) and sulfone (**10b**). Spectral data matched with that of literature. [17, 18]

Use of 4-Fluoro tagging in non-photocatalytic cross-coupling efforts

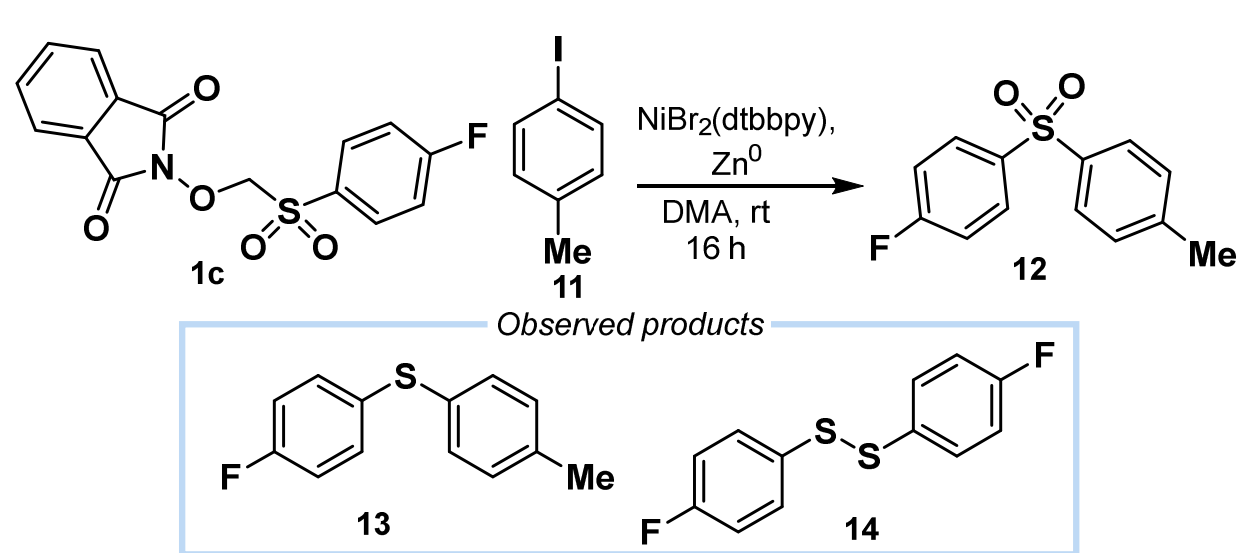

A sealed vial charged with **1c** (30 mg, 0.089 mmol, 1.5 equiv), 4-iodotoluene (13 mg, 0.060 mmol, 1 equiv), $NiBr_2$(dtbbpy) (4 mg, 0.009 mmol, 10 mol%), and Zn^0 (8 mg, 0.12 mmol, 2 equiv) was evacuated and backfilled with N_2 (x3). DMA (0.4 M) was then added via syringe. The resulting mixture was stirred at room temperature for 16 h. Upon completion, 4-fluoroacetophenone was added as an internal standard and 500 µLof the reaction mixture was transferred directly to a NMR tube and diluted with $CDCl_3$. ^{19}F NMR of the crude reaction mixture was analyzed to identify formation of the sulfide (**13**) and disulfide (**14**). The desired sulfone (**12**) was not obtained. Spectral data matched with that of literature. [13]

Full spectra of optimization trials included below for transparency. Entry 1, standard conditions. Entry 2, at 60°C instead of room temperature. Entry 3, DMSO instead of DMA. Entry 4, Bphen instead of dtbbpy. 4-fluoroacetophenone referenced to δ -105.4 ppm.

$^{19}F\{^1H\}$ NMR ($CDCl_3$, 376 MHz)

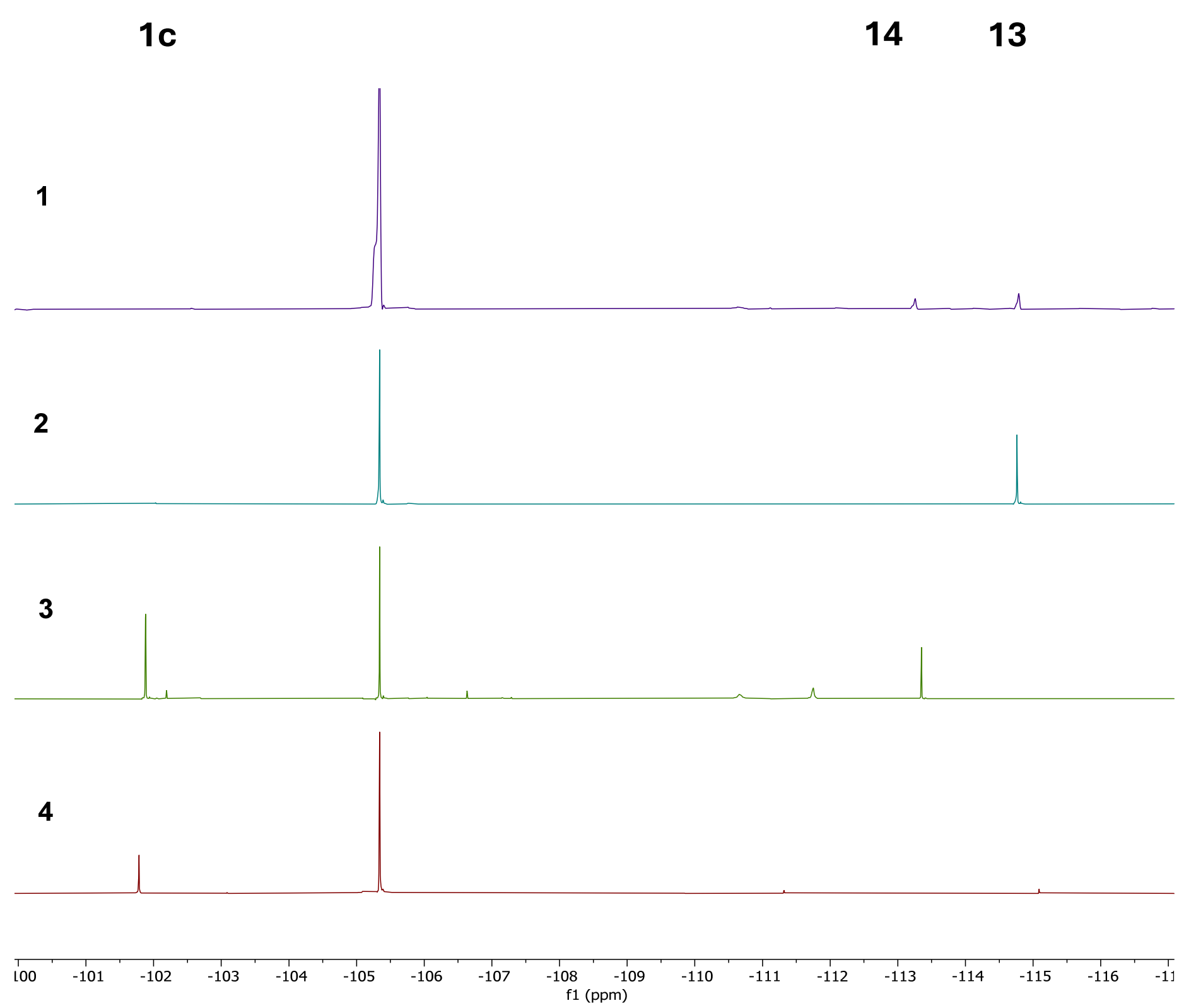

Table S5: ^{19}F NMR yields from Figure 5.

**Entry**	**^{19}F NMR yields**		
	**1c**	**14**	**13**
1	n.d.	9%	7%
2	n.d.	n.d.	41%
3	72%	36%	n.d.
4	23%	5%	5%

Use of 4-Fluoro tagging in Aniline Sulfonylation

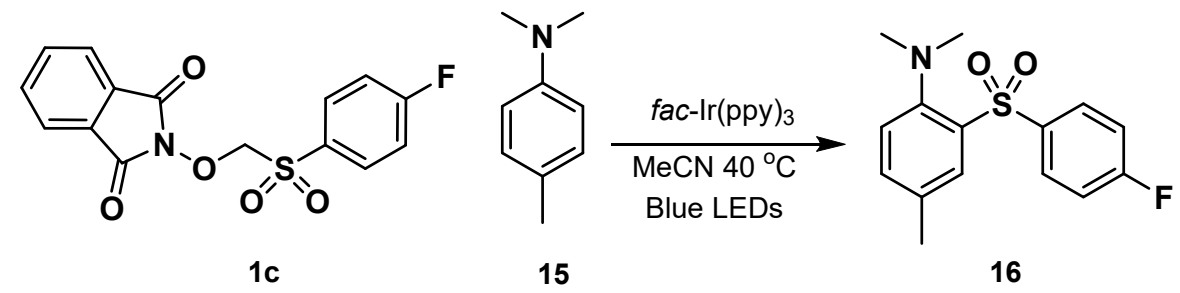

A sealed via charged with N,N,4-trimethylaniline (13 µL, 0.09 mmol, 1 equiv), **1c** (60.4 mg, 0.18 mmol, 2 equiv), and *fac*-Ir(ppy)$_3$ (1.2 mg, 2 mol%). The vial was then evacuated and backfilled with N_2 (x3), and degassed MeCN (0.1 M) was then added to the vial via syringe. The reaction was irradiated at 40°C for 16 h at approximately 5.0 cm from a Kessil blue LED (λ_{max}= 440 nm, 100% intensity). Upon completion, 4-fluoroacetophenone (1 equiv) was added to the crude reaction mixture as an internal standard, and 500 µLof the sample was transferred directly to a NMR tube and diluted with $CDCl_3$. ^{19}F NMR of the crude reaction mixture was analyzed to identify formation of the byproducts listed below. The reaction mixture was then purified using silica gel (eluting with ethyl acetate/hexanes) to confirm validity of initial assignments via other spectral data.

*Note: These conditions are unoptimized, reported here to illustrate the formation of disproportionation products. Optimized conditions and full scope of obtained aniline sulfonylation have been reported in the literature. [14]

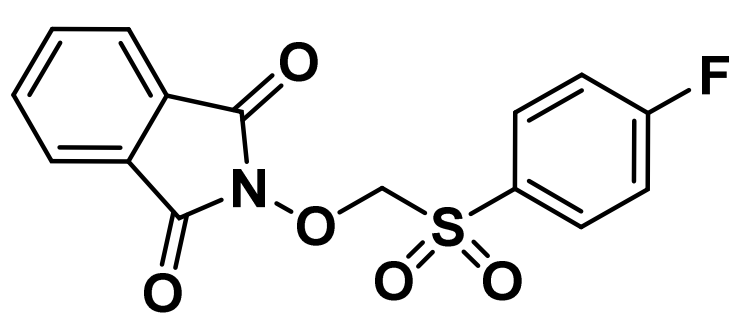

1c

Spectral data matched with that of literature. [14]

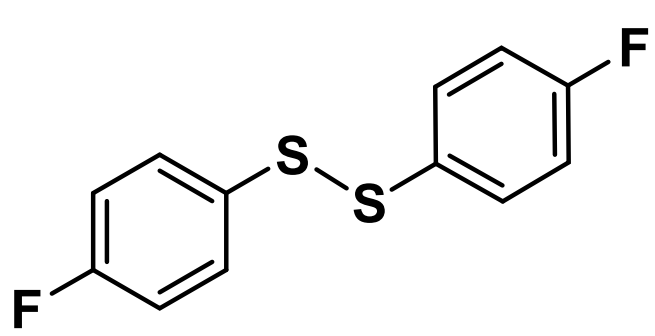

14

Spectral data matched with that of literature. [1]

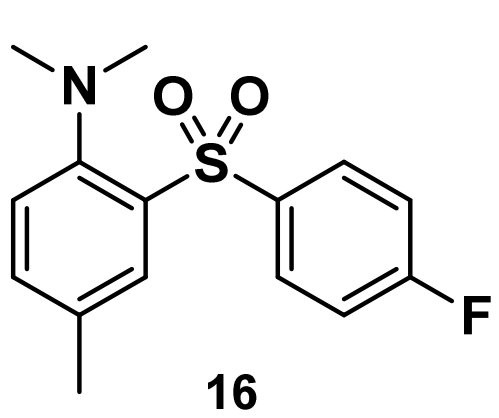

16

Spectral data matched with that of literature. [14]

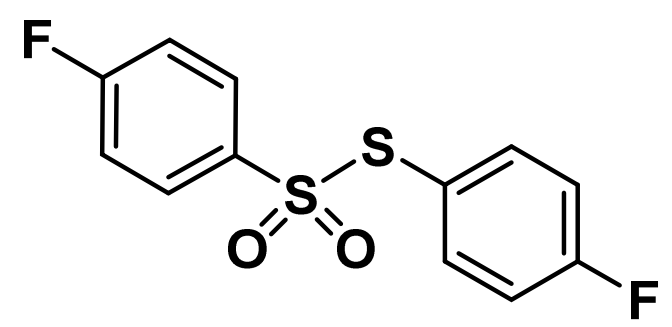

17

Spectral data matched with that of literature. [1]

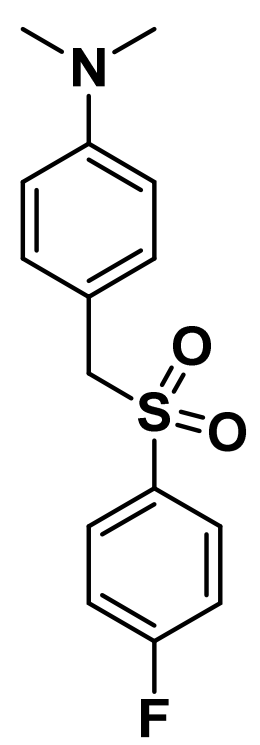

18

Yellow solid. **^{1}H NMR** (400 MHz, $CDCl_3$) δ 7.65 – 7.59 (m, 2H), 7.11 (t, *J* = 8.6 Hz, 2H), 6.94 – 6.88 (m, 2H), 6.61 – 6.55 (m, 2H), 4.21 (s, 2H), 2.94 (s, 6H). **^{13}C {1H} NMR** (101 MHz, $CDCl_3$) δ 165.9 (d, *J* = 255.7 Hz), 150.8, 134.4 (d, *J* = 3.2 Hz), 131.7 (d, *J* = 4.7 Hz), 131.7, 116.2 (d, *J* = 22.6 Hz), 114.8, 112.3, 62.8, 40.4. **^{19}F {^{1}H} NMR** (376 MHz, $CDCl_3$) δ -104.1. **HRMS** (ESI-TOF) m/z: $[M+H]^+$ calc'd for $C_{15}H_{17}FNO_2S$ 294.0964; found 294.0984.

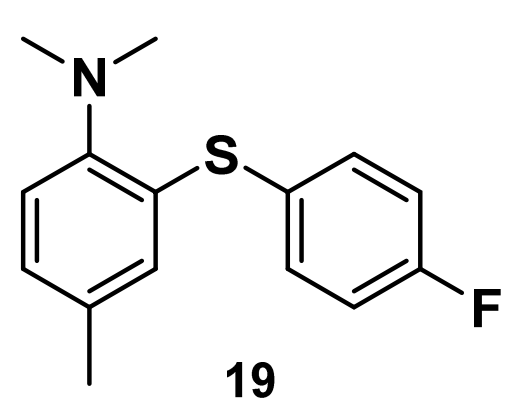

19

Yellow oil. **^{1}H NMR (400 MHz, $CDCl_3$)** δ 7.48 – 7.41 (m, 2H), 7.10 – 7.04 (m, 2H), 7.02 (d, *J* = 8.06 Hz, 1H), 6.94 (dd, *J* = 8.06, 2.03 Hz, 1H), 6.59 (d, *J* = 2.01 Hz, 1H), 2.77 (s, 6H), 2.16 (s, 3H).**^{13}C {1H} NMR** (101 MHz, $CDCl_3$) δ 162.9 (d, *J* = 248.0 Hz), 149.1, 135.9 (d, *J* = 8.2 Hz), 133.8, 133.3, 129.2 (d, *J* = 3.4 Hz), 129.0, 127.3, 119.6, 116.6 (d, *J* = 21.8 Hz), 44.8, 20.9. **^{19}F {^{1}H} NMR** (376 MHz, $CDCl_3$) δ -113.5. **HRMS** (ESI-TOF) m/z: $[M+H]^+$ calc'd for $C_{15}H_{17}FNS$ 262.1066; found 262.1075.

Real-time reaction monitoring

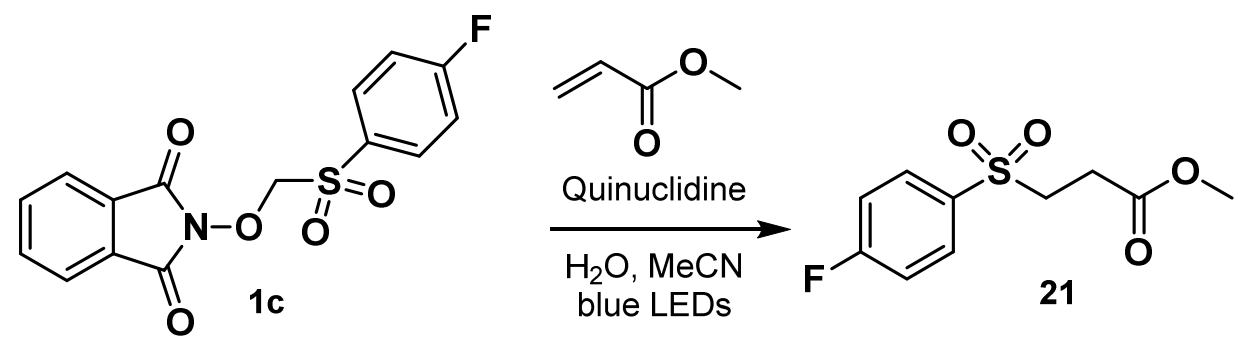

A 10 mL sealed vial was charged with quinuclidine (47 mg, 0.423 mmol, 1 equiv) and NHMP reagent **1c** (142 mg, 0.423 mmol, 1 equiv). The vial was then evacuated and backfilled with nitrogen (X3), and methyl acrylate (191 µL, 2.11 mmol, 5 equiv), water (76 µL, 4.23 mmol, 10 equiv), and degassed MeCN (4.2 mL, 0.1 M) were then added into the vial via syringe. Before irradiation, a 0.1 mL aliquot of the reaction mixture was removed and added directly to a NMR tube with 1.2 µL of 4-fluoroacetophenone added as an internal standard. The aliquot was then diluted with 0.4 mL $CDCl_3$. The reaction mixture was then irradiated at 40-50 °C (radiant heating) at approximately 5.0 cm from a Kessil blue LED (λmax = 440 nm, 100% intensity). A 0.1 mL aliquot of the reaction mixture was removed every 15 min over 1 h and diluted with 1.2 µL of 4-fluoroacetophenone and $CDCl_3$. Another aliquot was removed at the 2 hour mark. These samples were then analyzed by ^{19}F NMR, and the NMR yield of each species was calculated and plotted as a function of time to generate a reaction progress curve.

^{19}F{^{1}H} NMR ($CDCl_3$, 376 MHz)

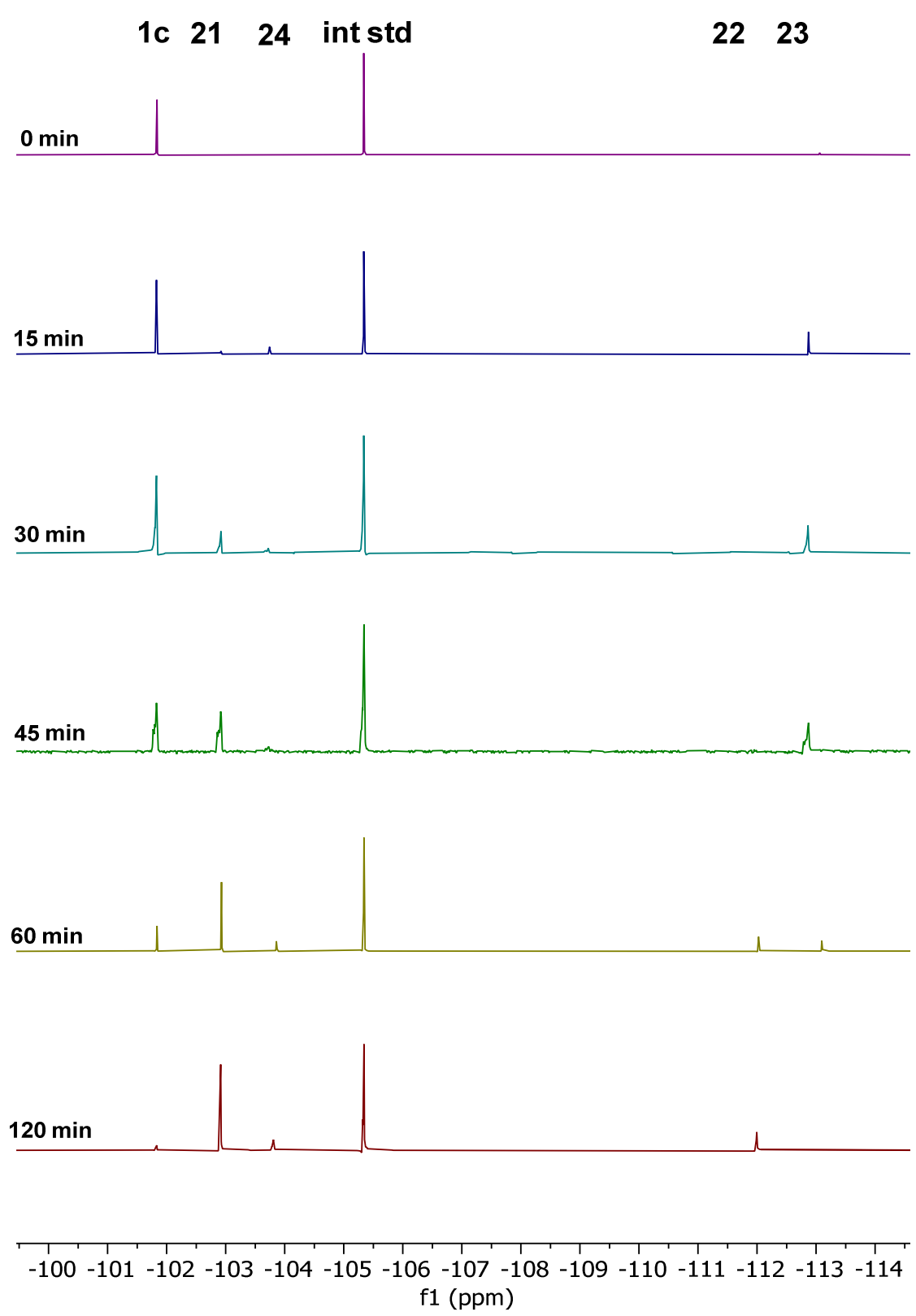

Characterization and Spectra of Isolated Compounds

^{1}H NMR ($CDCl_3$, 400 MHz)

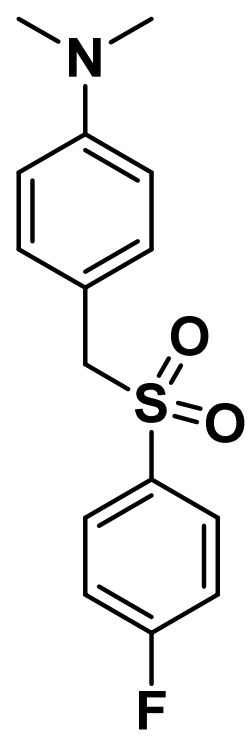

18

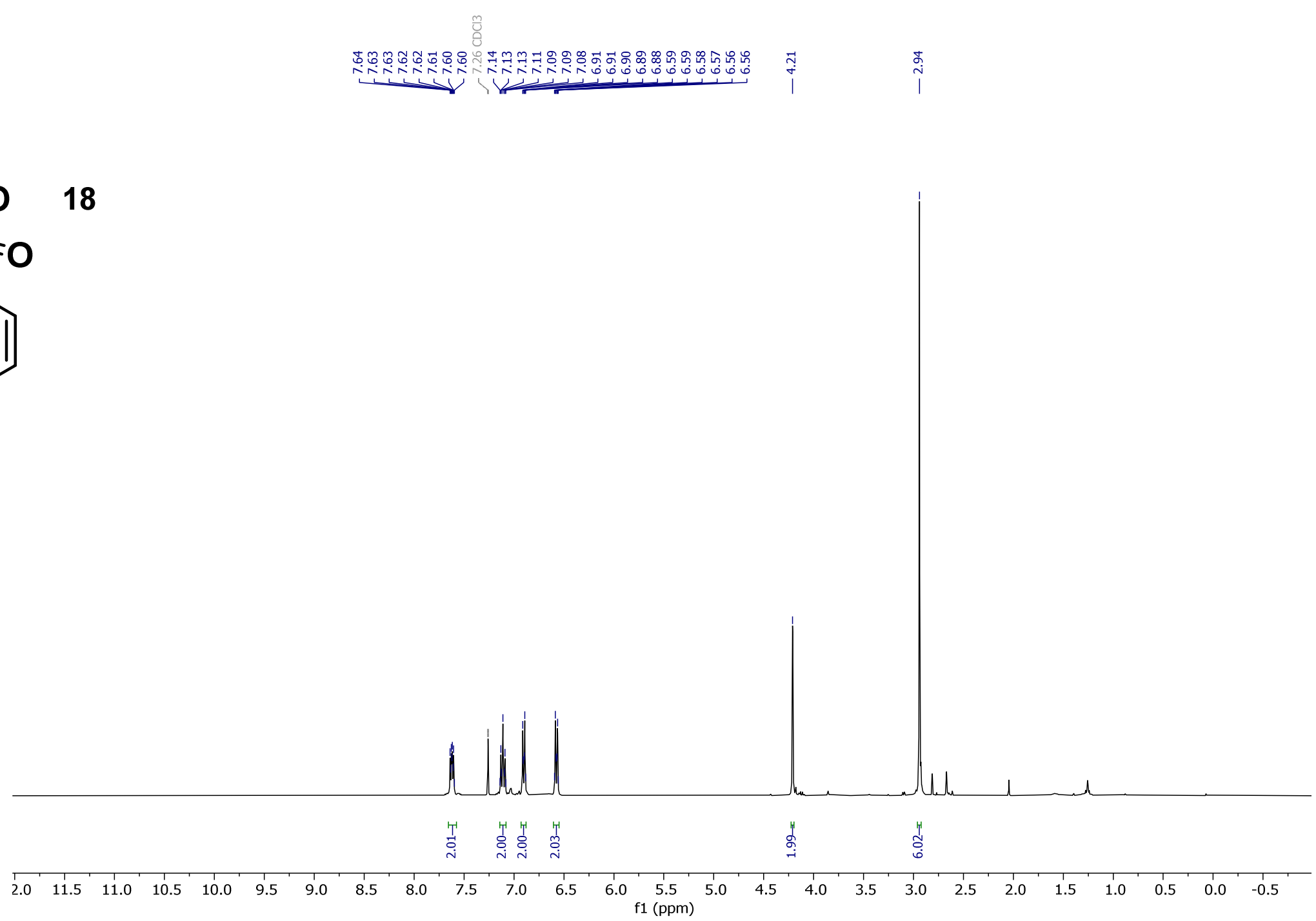

^{13}C{1H}NMR ($CDCl_3$, 101 MHz)

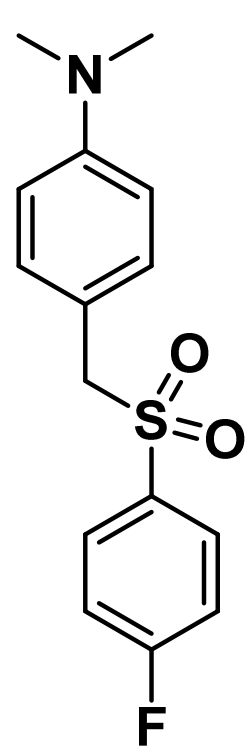

18

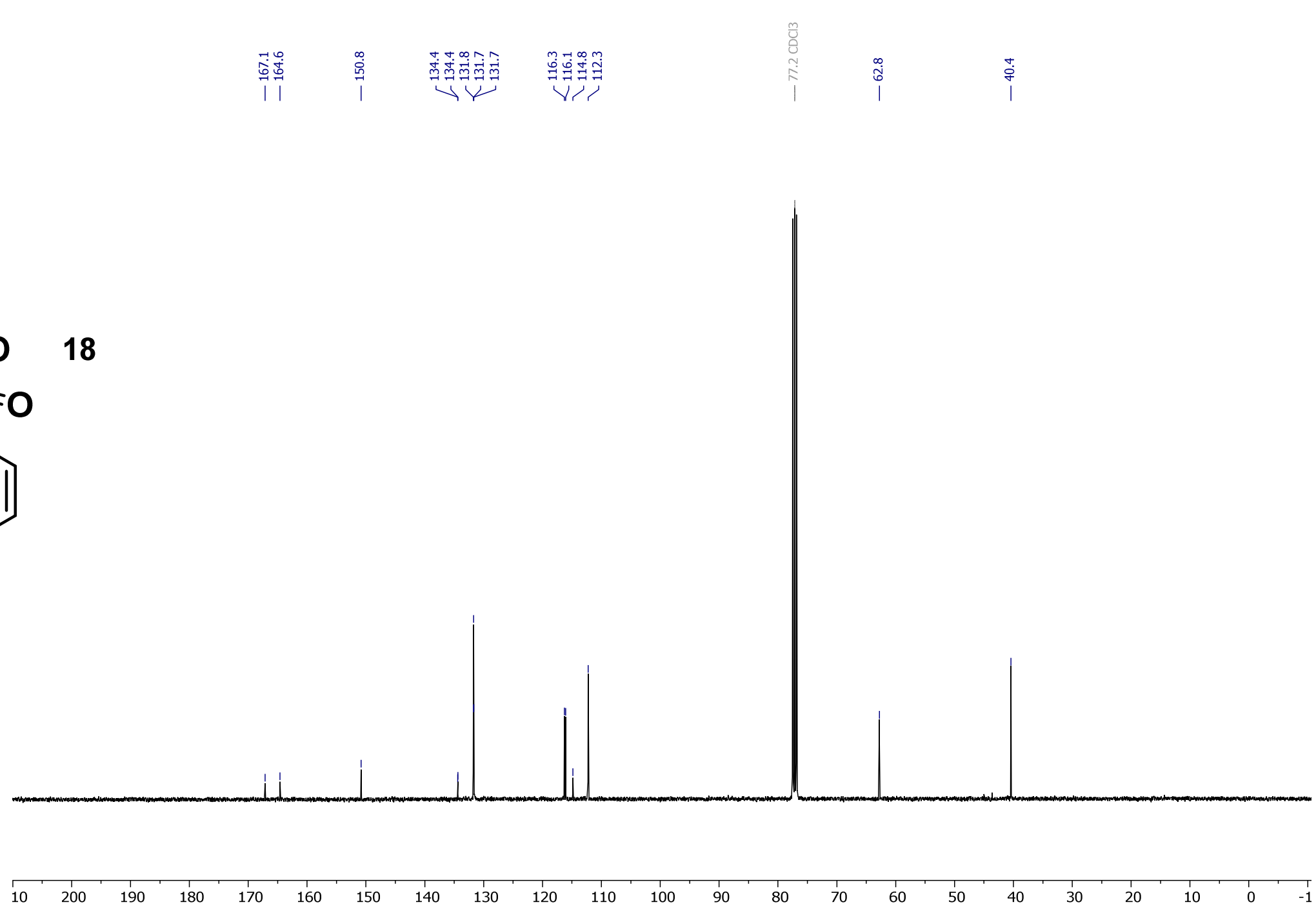

$^{19}F\{^{1}H\}$ NMR ($CDCl_3$, 376 MHz)

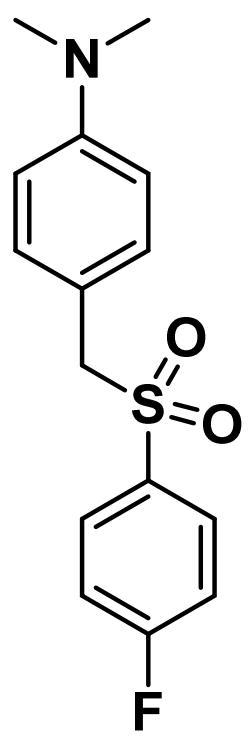

18

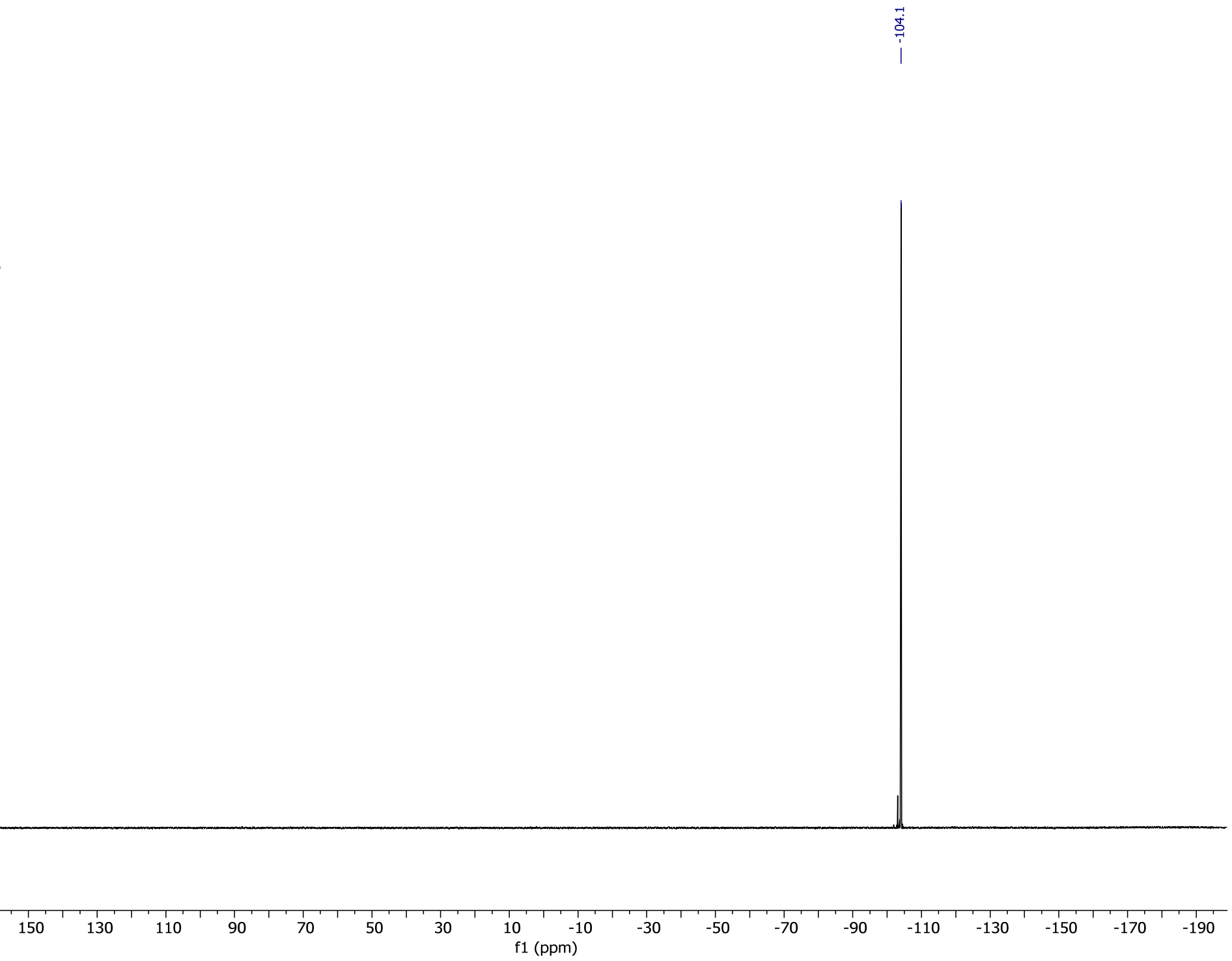

^{1}H NMR (CDCl3, 400 MHz)

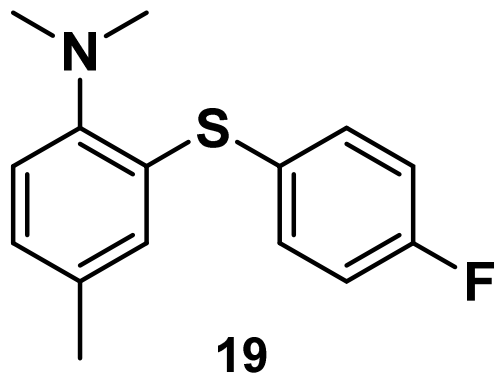

19

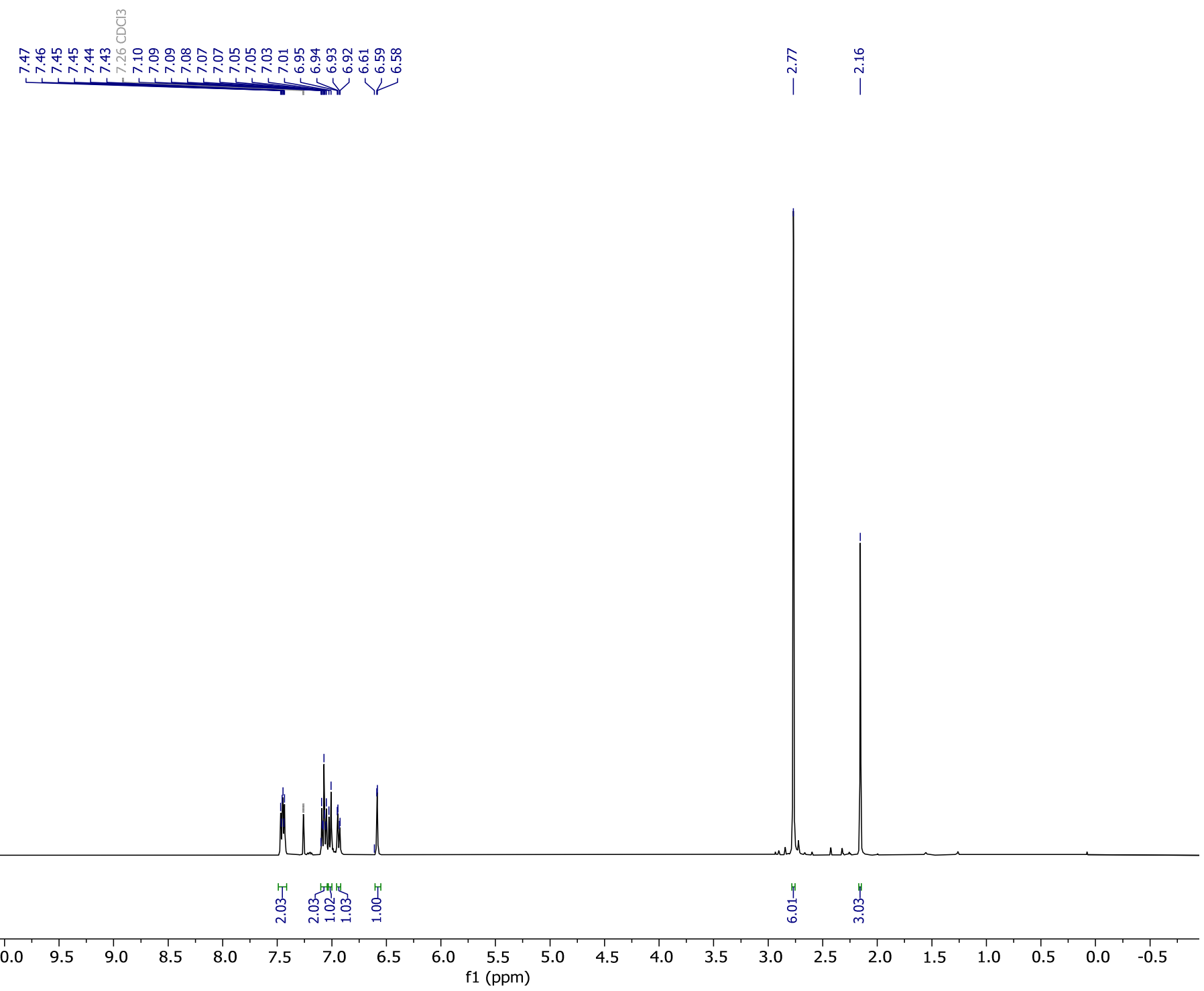

^{13}C{1H} NMR ($CDCl_3$, 101 MHz)

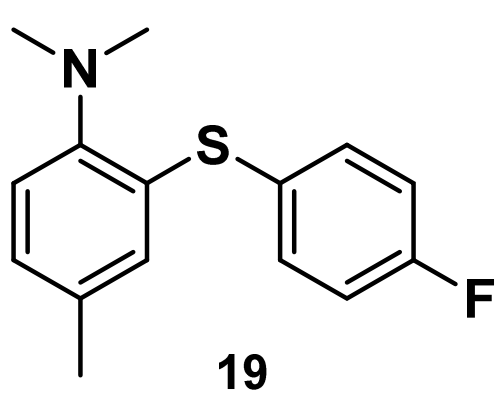

19

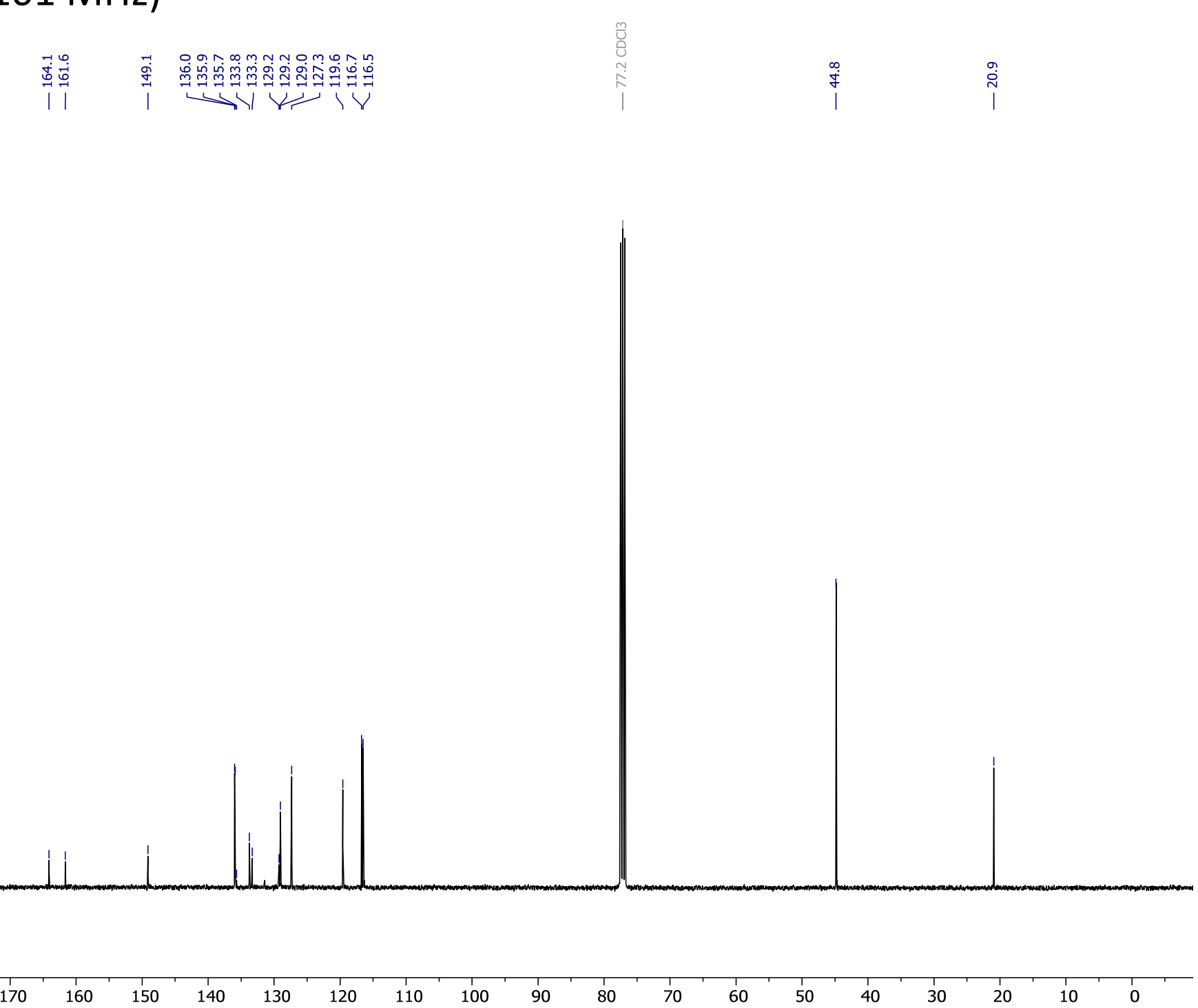

^{19}F{^{1}H} NMR ($CDCl_3$, 376 MHz)

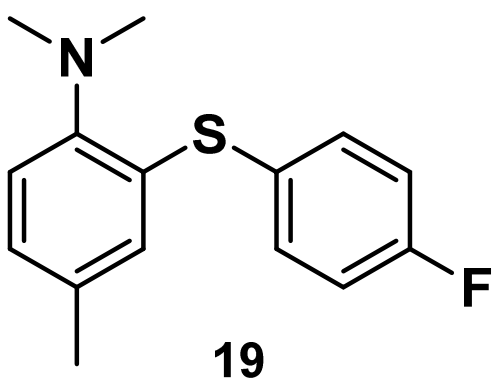

19

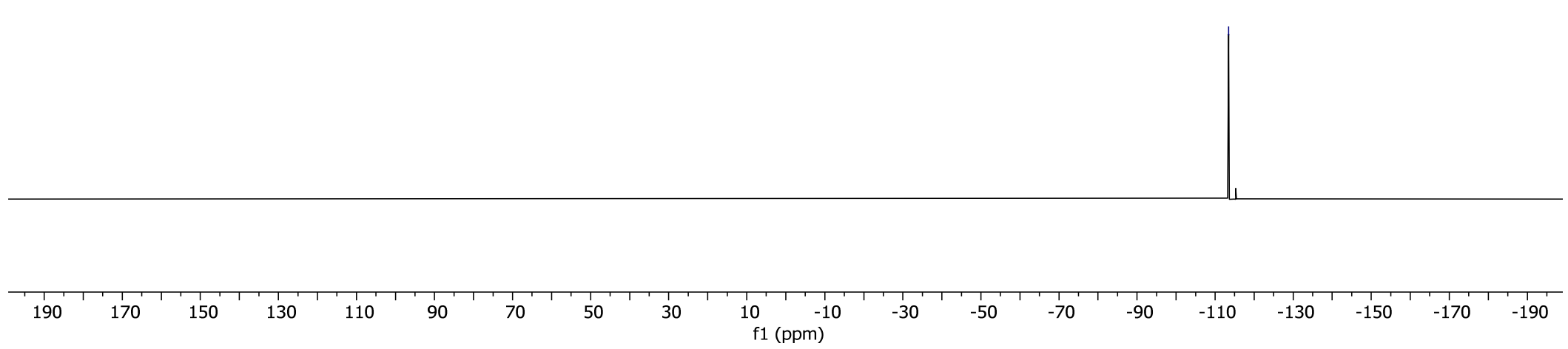

References

[1] Y. Zheng, F. L. Qing, Y. G. Huang, X. H. Xu, "Tunable and Practical Synthesis of Thiosulfonates and Disulfides from Sulfonyl Chlorides in the Presence of Tetrabutylammonium Iodide" *Adv. Synth. Catal.* **2016**, *358*, 3477-3481. 10.1002/adsc.201600633.

[2] T. I. Patel, M. L. Williams, Y. Chi, R. Laha, M. J. Moschitto, "Streamlining SuFEx Inhibitor Development: A Unified Approach Using Photolabile and Orthogonal Sulfinate Protecting Groups" *Angewandte Chemie International Edition* **2025**, *64*, e202505984. https://doi.org/10.1002/anie.202505984.

[3] S. Greed, E. L. Briggs, F. I. M. Idiris, A. J. P. White, U. Lücking, J. A. Bull, "Synthesis of Highly Enantioenriched Sulfonimidoyl Fluorides and Sulfonimidamides by Stereospecific Sulfur–Fluorine Exchange (SuFEx) Reaction" *Chemistry – A European Journal* **2020**, *26*, 12533-12538. https://doi.org/10.1002/chem.202002265.

[4] H. Zhu, Y. Shen, Q. Deng, T. Tu, "Copper-catalyzed electrophilic amination of sodium sulfinates at room temperature" *Chem Commun (Camb)* **2015**, *51*, 16573-16576. 10.1039/c5cc06069a.

[5] N. W. Liu, S. Liang, N. Margraf, S. Shaaban, V. Luciano, M. Drost, G. Manolikakes, "Nickel-Catalyzed Synthesis of Diaryl Sulfones from Aryl Halides and Sodium Sulfinates" *Eur. J. Org. Chem.* **2018**, *2018*, 1208-1210. 10.1002/ejoc.201701478.

[6] S. Greed, O. Symes, J. A. Bull, "Stereospecific reaction of sulfonimidoyl fluorides with Grignard reagents for the synthesis of enantioenriched sulfoximines" *Chem. Commun. (Cambridge, U. K.)* **2022**, *58*, 5387-5390. 10.1039/D2CC01219G.

[7] F. Jin, Q. Hu, Q. Wang, J. Sun, K. Huang, C. G. Yan, Y. Han, H. Fei, L. Wang, "Synthesis of Sulfoxides by Palladium-Catalyzed Arylation of Sulfenate Anions with Aryl Thianthrenium Salts" *J. Org. Chem.* **2024**, *89*, 13319-13328. 10.1021/acs.joc.4c01418.

[8] S. M. Hell, C. F. Meyer, A. Misale, J. B. I. Sap, K. E. Christensen, M. C. Willis, A. A. Trabanco, V. Gouverneur, "Hydrosulfonylation of Alkenes with Sulfonyl Chlorides under Visible Light Activation" *Angewandte Chemie International Edition* **2020**, *59*, 11620-11626. https://doi.org/10.1002/anie.202004070.

[9] N. M. McNeil, C. McDonnell, M. Hambrook, T. G. Back, "Oxidation of Disulfides to Thiolsulfinates with Hydrogen Peroxide and a Cyclic Seleninate Ester Catalyst" *Molecules* **2015**, *20*, 10748-10762. 10.3390/molecules200610748.

[10] W. P. Chappell, M. Favie, G. M. Sammis, "Thionyl fluoride as a sulfur(IV) SuFEx hub for the efficient syntheses of sulfinamides and sulfinate esters" *Chem Commun (Camb)* **2024**, *60*, 9765-9768. 10.1039/d4cc02876g.

[11] B. Zhao, Y. Fu, R. Shang, "Oxalic Acid Monothioester for Palladium-Catalyzed Decarboxylative Thiocarbonylation and Hydrothiocarbonylation" *Org. Lett.* **2019**, *21*, 9521-9526. 10.1021/acs.orglett.9b03701.

[12] S. Liu, Z. Qi, Z. Zhang, B. Qian, "Iodine/Manganese Dual Catalysis for Oxidative Dehydrogenation Coupling of Amines with Thiols" *Org. Lett.* **2019**, *21*, 7722-7725. 10.1021/acs.orglett.9b02545.

[13] S. C. Lee, H. H. Liao, A. Chatupheeraphat, M. Rueping, "Nickel-Catalyzed C-S Bond Formation via Decarbonylative Thioetherification of Esters, Amides and Intramolecular

Recombination Fragment Coupling of Thioesters" *Chemistry* **2018**, *24*, 3608-3612. 10.1002/chem.201705842.

[14] T. I. Patel, M. L. Williams, J. M. Linder, S. S. Shah, M. J. Moschitto, "Sulfonylation of Anilines through Photocatalytic Activation of N-Hydroxymethylphthalimide Sulfones" *ACS Catalysis* **2025**, *16*, 744-751. 10.1021/acscatal.5c07507.

[15] H. Yue, C. Zhu, M. Rueping, "Cross-Coupling of Sodium Sulfinates with Aryl, Heteroaryl, and Vinyl Halides by Nickel/Photoredox Dual Catalysis" *Angew Chem Int Ed Engl* **2018**, *57*, 1371-1375. 10.1002/anie.201711104.

[16] I. H. Lindenmaier, R. C. Richter, M. Schier, M. Strobele, I. Fleischer, "Nickel-Catalyzed C-S Cross-Coupling Enhanced by Mechanochemistry" *Acs Catalysis* **2025**, *15*, 17871-17881. 10.1021/acscatal.5c04516.

[17] S. Zhong, Z. Zhou, F. Zhao, G. Mao, G. J. Deng, H. Huang, "Deoxygenative C-S Bond Coupling with Sulfinates via Nickel/Photoredox Dual Catalysis" *Org. Lett.* **2022**, *24*, 1865-1870. 10.1021/acs.orglett.2c00478.

[18] J. Guilbaud, M. Labonde, A. Selmi, M. Kammoun, H. Cattey, N. Pirio, J. Roger, J. C. Hierso, "Palladium-catalyzed heteroaryl thioethers synthesis overcoming palladium dithiolate resting states inertness: Practical road to sulfones and H-sulfoximines" *Catal. Commun.* **2018**, *111*, 52-58. 10.1016/j.catcom.2018.03.025.

[19] T. Schoenberger, R. Bachmann, N. Gerhardt, J. Panzer, K. Meyer, M. Romoth, J. Teipel, A. Scharinger, M. Weber, T. Kuballa, M. Maiwald, S. Esslinger, C. Fauhl-Hassek, B. Horn, J. Riedl, R. Becker, E. Annweiler, M. Meier, M. Ohmenhäuser, M. Stoyke, *Guide to NMR Method Development and Validation – Part I: Identification and Quantification (update 2023)*, **2023**.
